# Characterizing Population Pharmacokinetics of Vatiquinone in Healthy Volunteers and Patients with Friedreich’s Ataxia

**DOI:** 10.3390/ph18091339

**Published:** 2025-09-06

**Authors:** Yongjun Hu, Lan Gao, Lucy Lee, Jonathan J. Cherry, Ronald Kong

**Affiliations:** PTC Therapeutics, Warren, NJ 07059, USA; lgao@ptcbio.com (L.G.); llee@ptcbio.com (L.L.); jcherry@ptcbio.com (J.J.C.); rkong@ptcbio.com (R.K.)

**Keywords:** vatiquinone, population pharmacokinetic modelling analysis (PopPK), patients with Friedreich’s ataxia (FA), healthy volunteers

## Abstract

**Introduction:** Vatiquinone is a first-in-class, small molecule designed to maintain mitochondrial function in the disorders like Friedreich’s ataxia (FA). Vatiquinone inhibits 15-lipoxygenase, consequently decreasing oxidative stress and neuroinflammatory response pathways. **Methods**: Population pharmacokinetic modeling analysis was conducted to characterize vatiquinone pharmacokinetic profiles in healthy volunteers and patients and explore the effects of covariates on vatiquinone exposures. **Results:** A two-compartment model with parallel zero- and first-order absorption was developed and verified. The values of essential parameters were: absorption fraction through the first-order process, 74.4%; absorption rate constant, 0.20 h^−1^; delay time, 2.79 h; zero-order absorption duration, 6.03 h; apparent volume of distribution, 180.75 L for the central and 4852.69 L for the peripheral compartment; and apparent clearance, 162.72 L/h. Strong CYP3A4 inducers could reduce exposure by 50%; strong CYP3A4 inhibitors could increase it by 252%. Vatiquinone exposure was 19% lower in patients with Friedreich’s ataxia versus healthy volunteers. A medium-fat meal increased exposure up to 25-fold versus a fasted status. Body weight and body mass index had significant clinical relevance to exposures. **Conclusions:** A two-compartment model effectively described the pharmacokinetic profiles of vatiquinone after oral administration. Covariates significantly impacted exposures, including body weight, meals, disease status, comedications and body mass index.

## 1. Introduction

Friedreich’s ataxia (FA) is a rare, inherited, progressive neuromuscular disorder that primarily affects the nervous system and heart, resulting in severe neurological symptoms, such as muscle weakness, sensory loss and hypertrophic cardiomyopathy, that sometimes suddenly lead to death. FA affects approximately 1/40,000 people in the United States [1]. FA is caused by mutations in the frataxin (*FXN*) gene that result in decreased expression of FXN, a mitochondrial protein necessary for the formation of iron–sulphur clusters to maintain proper mitochondrial function. Reduced FXN levels result in iron dysregulation, mitochondrial dysfunction, and high levels of oxidative stress [2]. Recent work has confirmed the link between oxidative stress and the role of ferroptosis in FXN pathology and established the reduction in ferroptosis as a rational and promising approach to treat FA [3,4,5,6]. While the first treatment was recently approved for the treatment of patients with FA aged 16 years and older by the FDA and EMA, there are still unmet medical needs for FA patients, particularly those under 16 years of age.

Vatiquinone, also known as PTC743, is a first-in-class, selective inhibitor of the enzyme 15-lipoxygenase (15-LO), which is a key regulator of fatty acid oxidation and the ferroptosis pathway with a central role in the pathogenesis of neurological diseases, including FA and other mitochondrial diseases [7]. 15-LO is activated when cells are under oxidative stress conditions. In the presence of free iron and oxygen free radicals, 15-LO converts polyunsaturated fatty acids to lipid hydroperoxides, such as 15(*S*)-hydroperoxyeicosatetraenoic acid (15[S]-HpETE) [8]. Accumulation of lipid hydroperoxides in the cell and within the cellular membrane compromises cellular integrity, triggering ferroptotic cell death [9]. Vatiquinone’s 15-LO inhibition is intended to prevent accumulation of lipid hydroperoxides, depletion of glutathione, proinflammatory signaling and ferroptosis, which are underlying pathologies of FA and other diseases associated with mitochondrial dysfunction. As a potential therapeutic agent, vatiquinone has undergone extensive evaluation for its clinical safety and efficacy in various populations, including adult healthy volunteers and pediatric and adult patients with FA and other mitochondrial diseases.

A population pharmacokinetic (PopPK) modeling analysis was conducted to acquire a better understanding of the pharmacokinetic (PK) profiles of vatiquinone in different populations as well as potential intrinsic and extrinsic factors leading to inter-participant variability. This analysis characterized the plasma concentration–time profiles of orally administered vatiquinone using PK data collected from eight Phase I/II/III studies in healthy adult volunteers and pediatric and adult patients with FA or other mitochondrial diseases. The PopPK model could be utilized to simulate various dose regimens to guide dose selection for future clinical studies with vatiquinone.

## 2. Results

### 2.1. Pharmacokinetic (PK) Sampling and Demographics of Analysis Population

The numbers of participants and PK samples in the NONMEM ready dataset, stratified by study, are summarized in Table 1. The dataset included a total of 343 unique participants and 4608 quantifiable PK samples.

Categorical covariates for the participants are summarized in Supplementary Table S1. In the study population, 48.1% of participants were male and 51.9% were female; 33.8% were healthy volunteers, 50.4% were patients with FA, and 15.7% were patients with other mitochondrial diseases; 42.0% were pediatrics along with 29 patients with <7 years of age and 58.0% were adults in total of participants. More than half of participants were White (81.3%), and the rest were Black or African American (10.2%), Asian (3.5%), and of unknown race (5.0% participants did not report race).

In continuous covariate data, the median age was 20 years old (range: 1–67 years old) and median baseline body weight was 58.6 kg (range: 6.3–119 kg). The median dose was 400 mg (range 120–1400 mg); more summarized results are shown in Supplementary Table S2.

### 2.2. Exploratory Data Analysis of PK Concentrations

**Figure 1:**
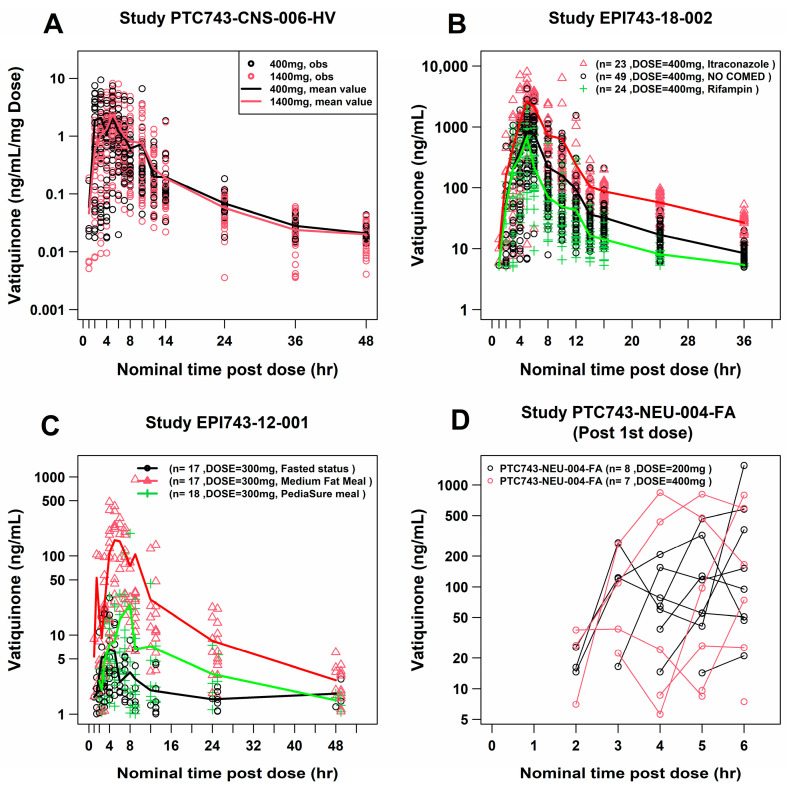
Selected concentration–time profiles of vatiquinone following oral single doses. Dose normalized vatiquinone plasma concentration–time profile showed a decent superimposition at following oral administration, once daily in the study PTC743-CNS-006-HV (**A**), CYP3A4 inducer and inhibitor effect on vatiquinone exposures in the study EPI743-18-002 (**B**), food effect on vatiquinone exposures in the study EPI743-12-001 (**C**), and time delay absorption process in the study PTC743-NEU-004-FA (**D**).

A general plasma vatiquinone concentration distribution and concentration–time profiles by study are depicted in Supplementary Figure S1. Using intensive PK sampling data points after single dose administration illustrates the congruence of dose-normalized curves in Study PTC743-CNS-006-HV (Figure 1A), which strongly supports the notion of a dose-proportional PK profile. This finding aligns with the conclusions drawn from the dose linearity assessment, as detailed in supplementary non-compartmental analysis (NCA) (Supplementary Figure S2 and Table S3), and the consistency holds true across a wide range of administered doses spanning from 200 to 1400 mg.

Moreover, as elucidated by the PK profiles presented in Figure 1B,C, it is evident that vatiquinone plasma concentrations are notably influenced by extrinsic factors, particularly the consumption of food, e.g., PediaSure^®^ liquid, and the simultaneous administration of specific comedications, such as the CYP3A4 inhibitor, itraconazole, and the CYP3A4 inducer rifampin. This influence becomes conspicuously discernible when these conditions are juxtaposed against the reference control.

Additionally, an intriguing observation pertains to a delayed absorption pattern, manifesting as a visible lag of 2–3 h following the initial dose (Figure 1D). This phenomenon has been observed in Study PTC743-CNS-006-HV and consistently confirmed by parallel investigations in Study PTC743-NEU-004-FA (Supplementary Figure S3), where their PK profiles clearly showed the time-delayed absorption.

### 2.3. PopPK Modeling Analysis

Since the participants included youth and adult participants, and there were data from clinical food effect and drug–drug interaction studies, the baseline body weight, meal status, and comedication (CYP3A4 inhibitor and inducer) were integrated in the base model. Additionally, after backward elimination, three additional covariates, including disease FA on relative bioavailability (FK0), disease FA on apparent clearance (CL), and body mass index (BMI) on clearance (CL), were retained. Collectively, in the final PopPK model, the PK parameters absorption rate constant (Ka), absorption fraction (FK0), clearance (CL), and volume of distribution (V) are described below:

$${Ka}_{i}=TVKa\cdot exp({\eta_{i}}^{Ka})$$

$${FK0}_{i}=TVFK0\cdot e^{{{LQD}_{i}\cdot \theta }_{12}}\cdot e^{{FST}_{i}\cdot \theta_{13}}\cdot (1+{FA}_{i}\cdot \theta_{16})$$

$${CL}_{i}=TVCL\cdot \left(1+{FA}_{i}\cdot \theta_{15}\right)\cdot e^{{{ITR}_{i}\cdot \theta }_{10}}\cdot e^{{RFM}_{i}\cdot \theta_{11}}\cdot {\left[\frac{{BWT}_{i}}{65}\right]}^{\theta_{14}}{\left[\frac{{BMI}_{i}}{21.6}\right]}^{\theta_{17}}\cdot exp({\eta_{i}}^{CL})$$

$$V_{i}=TVV\cdot {\left[\frac{{BWT}_{i}}{65}\right]}^{1}\cdot exp({\eta_{i}}^{V})$$

where TVKa, TVFK0, TVCL, and TVV denote the typical value of Ka (absorption rate constant), FK0 (fraction of absorption through first-order absorption, F1), CL (apparent clearance), and V (apparent volume distribution of central compartment). Ka_i_, FK0_i_, CL_i_, and V_i_ denote the corresponding parameter for the ith individual. BMI_i_ denotes the BMI value for the ith individual; FA_i_ is set to 1 for participants with FA and is set to 0 for healthy volunteers. With respect to the feeding condition (medium-fat meal: food with ≥25% of calories from fat), LQD_i_ assumes a value of 1 when a liquid PediaSure^®^ meal is ingested, while FST_i_ is set to 1 in the fasted state. In the absence of these conditions, both LQD_i_ and FST_i_ are designated as 0, signifying the reference medium-fat meal condition (food with ≥25% of calories from fat). ITR_i_ is set to 1 when there is a concomitant administration of itraconazole and RFM_i_ equals 1 when rifampin is co-administered; otherwise, ITR_i_ and RFM_i_ are assigned a value of 0, representing the reference condition of vatiquinone monotherapy, respectively. BWT_i_ denotes the baseline body weight for the ith individual. η_i_ denotes random effect for the ith individual. The median BMI value of the population is 21.6 kg/m^2^, while the reference body weight value is set to 65 kg in the population.

Parameter estimates for the final model are summarized in Table 2. Goodness-of-fit (GOF) plots for the final PK model are included in Supplementary Figure S4. In the final model, a total of zero observations in the dataset were identified as potential outliers according to the criterion of |CWRES| > 5 described in Section 4.6.

### 2.4. Visual Predictive Check (VPC) and Forest Plots

**Figure 2:**
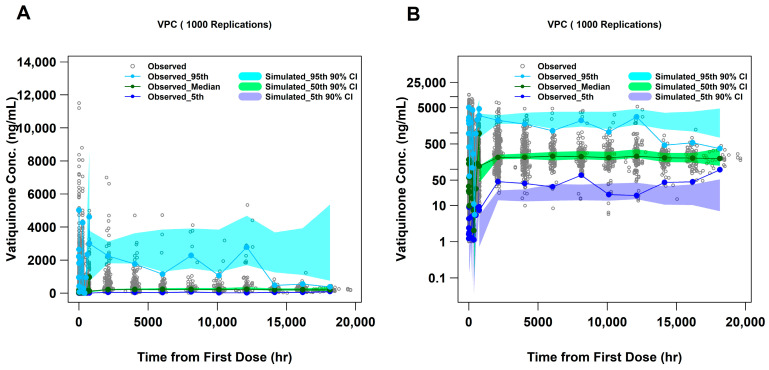
Visual predictive check (VPC) plots for the final model. A total of 1000 simulations were implemented with fixed PK parameters in the final model, a linear scale (**A**) and a log scale (**B**). The shadow indicates the 90% confidence interval (CI) for the predicted 5th, 50th, and 95th percentiles of vatiquinone concentration. CI: confidence interval; Conc.: Concentration; PK: pharmacokinetic.

VPC plots, using a linear and a log scale, are presented in Figure 2. Additional VPC plots stratified by study are included in Supplementary Figure S5. In these plots, observed data are generally contained within the simulated 90% confidence intervals, indicating that the final PopPK model adequately described vatiquinone concentration profiles and could be utilized for prediction.

**Figure 3:**
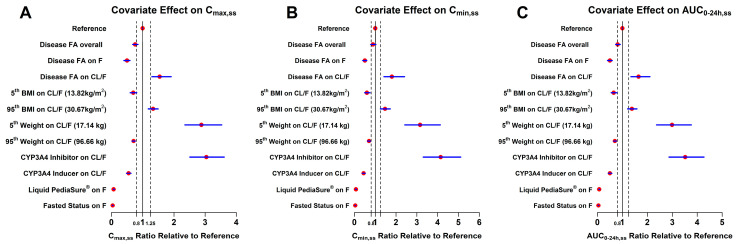
Influence of covariates on vatiquinone C_max,ss_ (**A**), C_min,ss_ (**B**), and AUC_0–24h,ss_ (**C**)_._ Red circles show the ratio of the median exposure metric under the test conditions compared to the reference. The blue line segments represent the corresponding 90% confidence interval. Test conditions for continuous covariates include the 5th and 95th percentiles among participants in the analysis dataset. Vertical dashed lines indicate the reference interval of 0.80–1.25. Simulations (N = 1000) were performed for virtual participants (one per test condition and reference), with parameter uncertainty incorporated using a smoothed parametric bootstrapping procedure based on the final model parameter estimates. Reference = healthy volunteer with 65 kg body weight, 21.6 kg/m^2^ BMI, no comedication, solution formulation drug with medium-fat meal (food with ≥25% of calories from fat) for oral administration. AUC_0–24h,ss_: area under the concentration–time curve from 0 to 24 h at steady state; BMI: body mass index; CL/F: apparent clearance; C_max,ss_: maximum concentration at steady state; C_min,ss_: minimum concentration at steady state; F: bioavailability or absorption fraction; FA, Friedreich’s ataxia.

Meanwhile, model-based simulations were performed to assess the impact of covariates on vatiquinone PK exposures at steady state condition. Figure 3 shows the C_max,ss_, C_min,ss_ and AUC_0–24h,ss_ after multiple doses of vatiquinone. In summary, among the covariates examined, the 5th percentile of body weight, the presence of itraconazole or rifampin, liquid PediaSure^®^ and fasted status had a notable impact on exposure, resulting in higher or lower C_max,ss_, C_min,ss_, and AUC_0–24h,ss_ when compared to the reference condition (Table 3). Overall, for patients with FA, PK exposures in terms of C_max,ss_, C_min,ss_, and AUC_0–24h,ss_, were 24%, 11%, and 19% lower, respectively, compared to the healthy volunteers (Table 3). Notably, the medium-fat meals (food with ≥25% of calories from fat) induced a substantial increase in PK exposure, with a 14-fold increase compared to the liquid PediaSure^®^ meal and a 25-fold increase compared to the fasted status. For the remaining covariates, the mean values and 90% confidence intervals of the test-to-reference ratios for C_max,ss_, C_min,ss_, and AUC_0–24h,ss_ are detailed in Table 3.

### 2.5. Internal Validation

**Figure 4:**
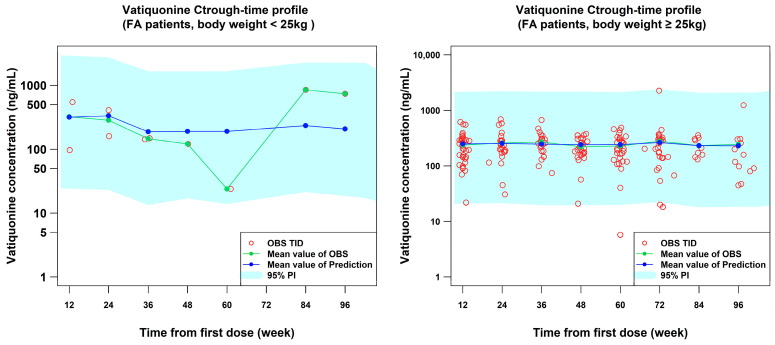
The comparison between observed and corresponding predicted C_trough_ for resampled participants from studies PTC743-NEU-003-FA and PTC743-NEU-005-FA, stratified by baseline body weight <25 kg (**left**) and ≥25 kg (**right**). C_trough_: trough concentration; FA: Friedreich’s ataxia; OBS: observed values; PI: prediction interval; TID: three times a day.

Simulations were performed to predict vatiquinone trough concentration (C_trough_ or C_min,ss_) in patients with FA using the PopPK final model with 1000 datasets, each dataset including 100 participants resampled from the PTC743-NEU-003-FA and PTC743-NEU-005-FA studies. As illustrated in Figure 4, the final PopPK model showcases the strength of its predictive capabilities in estimating the C_trough_ levels of vatiquinone in patients with body weight < 25 kg or ≥25 kg after three times a day (TID) treatments within the framework of the PTC743-NEU-003-FA and PTC743-NEU-005-FA studies (Supplementary Table S5). The percentage of PK observations stratified by body weight band and visit tabulated in Supplementary Table S4 shows that ≥93% of PK observations were contained within the 95% prediction interval for participants with ≥25 kg in body weight. Meanwhile, we also examined the model performance in predicting the concentration of participants with body weight < 25 kg. We found that 100% of PK observations were contained in the 95% prediction interval, indicating the final model is reliable to predict the vatiquinone concentration in younger pediatric patients with FA, although the participant number is limited (n = 5). Additionally, as shown in Supplementary Table S5, stratified by the subpopulation and body weight group, and Supplementary Table S6 by the subpopulation and age group, the proportion of % observed PK samples contained in the 95% prediction interval (PI) is ≥85.71%, which confirmed the reliability and robustness of the PopPK model.

Furthermore, as described by Sheiner and Beal [10], prediction-based parameters, prediction error (PE), and absolute prediction error (APE), along with most used metrics for evaluating the accuracy of models, including root mean square error (RMSE) as a measure of precision, mean prediction error (MPE) as a measure of bias, and mean absolute prediction error (MAPE) as a measure of accuracy, were calculated from typical predictions (PRED) and individual predictions (IPRED). The summary of PE, APE, RMSE, MPE, and MAPE, computed with the overall dataset [11], is shown in Table 4. The RMSE values are 0.84% for PRED and 0.90% for IPRED, both of which are far less than the threshold of 10%. The MPE values are 4.55% for PRED and 4.48% for IPRED, both of which fall at the edge of the prespecified criteria of ±10%. This indicates that the prediction error does not exceed 4.55% of observations on average. Additionally, the MAPE values are 11.90% for PRED and 14.90% for IPRED, both of which are below the 20% threshold. Overall, these results showed that the reliability, accuracy, and adequacy in the descriptive and predictive performance of the PopPK model were validated.

## 3. Discussion

An exploratory analysis of the vatiquinone plasma concentration–time profile revealed several key findings. The mean value of time to reach maximum concentration (T_max_) was approximately 6.5 h, with a range spanning from 0 to 24 h. This suggests that vatiquinone undergoes a moderately paced absorption process. Additionally, as illustrated in Figure 1D and Supplementary Figure S3, the increase in vatiquinone plasma concentration after dose administration was apparently delayed, typically occurring within a 2 to 3 h timeframe. This suggests a time-delay in the absorption process.

During the modeling analysis, we noticed that the duration of the zero-order absorption process was approximately 6 h (Theta 3, TK0 in Table 2). After oral administration, lipophilic drugs may be absorbed through a potential pathway via enterocytes into the lymphatic circulation, thereby gaining access to the systemic circulation. This absorptive process parallels a passive intestinal absorption process [12,13,14,15]. Vatiquinone, an analog compound of Vitamin E, which features a quinone ring that has an optimized redox potential and a lipid side chain. The partition coefficient (cLogP) is 7.8, indicating high lipophilicity and hydrophobicity. This is confirmed by its insolubility in aqueous solutions and solubility in organic solvents such as ester and alcohol (data on file). The lipophilic properties of vatiquinone suggests two potential absorptive pathways after absorption into the intestinal enterocyte. Vatiquinone can either enter the systemic circulation directly or associate with chylomicrons within fat cells, subsequently being secreted into the lymphatic circulation during the transit. Ultimately, these two parallel absorption processes result in vatiquinone entering the systemic circulation with an extended absorption duration of up to 6 h. On the other hand, as a lipophilic compound, vatiquinone tends to partition into lipid-rich environments, potentially slowing its dissolution in the aqueous gastrointestinal tract. Additionally, its molecular structure suggests moderate permeability, which may further delay absorption [16]. This theoretical interpretation supports the use of a two-compartment model with dual absorption processes to adequately describe the pharmacokinetic (PK) observations.

The shape of the terminal phase in the semi-log PK profile indicated that vatiquinone follows a consistent disposition profile across various dose levels, suggesting linear clearance kinetics (Figure 1B). Utilizing PK parameters derived through non-compartmental analysis, our linearity analysis indicated that vatiquinone exposure metrics, such as C_max_ and AUC_0–24h_ at Day 1, remained dose-proportional across a wide oral dose range of 200 to 1400 mg (Supplementary Figure S2). The power coefficient estimates for AUC_0–24h_ and C_max_ closely approached 1.0 (Supplementary Table S3) and the 95% confidence interval of power estimate g contained 1.0. Such a conclusion was also supported by the results of the β1 range in which the power estimate was included [17].

This model demonstrated adequate predictability in the terminal phase, with only minor overpredictions in the 95th percentile concentrations and slight underestimation in the 5th percentile concentration overall (Figure 2). Given the considerable dose range, extensive variability in drug concentrations, significant effects of food on bioavailability, and the broad time span of the studies, the final PopPK model still effectively aligned the mean values of observations (50th percentile) and predictions for the studies overall and for the individual studies (Supplementary Figure S5).

From the final PopPK model, typical PK parameters were as follows: a lag time for first-order absorption process (TLAG1) of 2.79 h, which is comparable with visual observations of the PK profile after a single or multiple dose (Figure 1D); a fraction of absorption of 74.4% through the first-order absorption process FK0 and 25.6% through the zero-order absorption, which had a duration of 6.03 h, consistent with vatiquinone’s lipophilic properties; a CL/F of 162.72 L/h; and a V/F of 180.75 L in the central compartment and 4852.69 L in the peripheral compartment. The high volume of distribution indicated the drug vatiquinone partitioned into the extravascular compartments of the body and had a remarkable impact from tissues. In addition, as presented in Table 2, patients with FA have a 50% reduction in relative bioavailability (FK0 and a 40% decrease in clearance compared to healthy volunteers. This results in an overall 19% decrease in AUC_0–24h,ss_ and a 24% decrease in C_max,ss_ compared to healthy volunteers. Table 3 and Figure 3 further demonstrate these findings for patients with FA disease. FA is a rare disease characterized by nonspecific symptoms. It is rare to identify children with FA. FA is typically diagnosed between 10 and 15 years of age. It is rare to see symptoms before 10 and in most cases, patients diagnosed with FA prior to 10 are siblings and were diagnosed through genetic testing, not appearance of symptoms. This put additional challenges for the enrollment of pediatric FA patients under the age of seven, as the limited number of FA patients in this study in this age group (n = 5) could influence the accuracy of modeling outcomes for younger patients. However, incorporating data from 29 pediatric patients under seven years of age (including 24 patients from the PTC743-MIT-001-EP), we assessed maturation and age as covariates on systemic clearance. No significant associations were observed with either covariate, indicating that the model remains robust in its ability to describe the observation for younger FA patients. Furthermore, the analysis dataset included one study conducted on patients with other mitochondrial diseases. While the effect of other mitochondrial diseases on vatiquinone exposures was examined, no significant differences were observed in PK exposures compared to healthy volunteers.

The robustness of the PopPK final model was confirmed using participants resampled from individuals with FA in studies PTC743-NEU-003-FA and PTC743-NEU-005-FA. As illustrated in Figure 4, the model-predicted C_trough_ closely aligns with the observed C_trough_ values from the clinical studies PTC743-NEU-003-FA and PTC743-NEU-005-FA. Furthermore, Supplementary Figure S4 depicts the goodness-of-fit (GOF) plots for the final PopPK model, showing a highly congruent overlay between population prediction (PRED), individual prediction (IPRED), and the observed data. Additionally, the performance evaluation values, such as RMSE for precision, MPE for bias, and MAPE for accuracy [18,19,20,21,22] demonstrated that there is only a 4.48% overestimate in PK exposure with a 90% confidence interval including 0; meanwhile, the RMSEs showing the prediction values are less than 1.0% from the “true” values of PK exposures in patients with FA (Table 4). Although the visual predictive check (VPC) study indicated a slight overprediction of peak concentration and underestimation of trough concentration (Figure 2), the 90% CI of the 50th percentile prediction fully contained the observed median values. Additionally, further descriptive statistics analysis and plots demonstrated that the 95% prediction intervals encompassed approximately 88.29 to 97.02% of the observed data points across the studies, as shown in Supplementary Table S7. Overall, these findings strongly support the conclusion that the final PopPK model adequately describes the observed data and is reliable and robust for predicting PK exposures in future studies. As a result, this model was utilized to simulate various dosing regimen scenarios.

## 4. Materials and Methods

### 4.1. Software

The population pharmacokinetics (PopPK) analyses were performed using the nonlinear mixed effects modeling methodology as implemented in NONMEM (Version 7.5, ICON Development Solutions, Ellicott City, MD, USA). Post-processing and graphical analysis of the outputs from the models were performed using R software (version 4.4.1 or higher, R Core Team, Vienna, Austria).

### 4.2. Ethics

All clinical studies were conducted in accordance with the Declaration of Helsinki. Protocols and consent forms received the requisite approvals from regulatory agencies and/or the local Independent Ethics Committee or Institutional Review Board at participating clinical sites prior to initiation of the studies. The clinical investigations were meticulously aligned with the established protocols and the principles of Good Clinical Practice. Prior to participation in clinical studies, all the participants provided the signed informed consent form.

### 4.3. Clinical Study Population and Design

The PopPK modeling analysis included vatiquinone plasma concentration–time data from eight Phase I/II/III studies: EPI743-12-001, EPI743-18-002, EPI-2010-006, PTC743-MIT-001-EP, PTC743-NEU-003-FA, PTC743-NEU-004-FA, PTC743-NEU-005-FA, and PTC743-CNS-006-HV. These studies included adult healthy volunteers and adult and pediatric patients with FA and other mitochondrial diseases, as summarized in Table 5.

### 4.4. PK Data Handling and Imputation

In general, this PopPK modeling analysis used as much of the available data as possible. The plasma concentration of vatiquinone was measured using a validated bioanalytical method previously outlined in the prior publication [23]. PK data from patients were included up to the final data collection point for that patient. Patients who experienced a change in dose were included with the recorded dose. Nonlinear mixed effects analysis accounted for individual fluctuations in dosing by using the actual dose amounts and times of administration. PK data missing due to assay sensitivity (i.e., below the quantification limit [BQL]), lost samples, lost dose records, or obviously erroneous records were excluded from the analysis if they could not be corrected based on a review of the data and site queries. During the final PopPK modeling analysis, BQL samples were handled using the “M1” method in which all BQL data were ignored [24].

### 4.5. Exploratory Data Analysis

Exploratory data analyses were performed to inform subsequent model development. Tabular summaries of observed PK samples and covariate data were generated to characterize the population. Concentration versus time plots with both linear and log-linear axes were generated to inform structural model development. These plots were stratified by relevant study conditions or covariates to assess trends in the data if needed.

### 4.6. PopPK Model Structure

**Figure 5:**
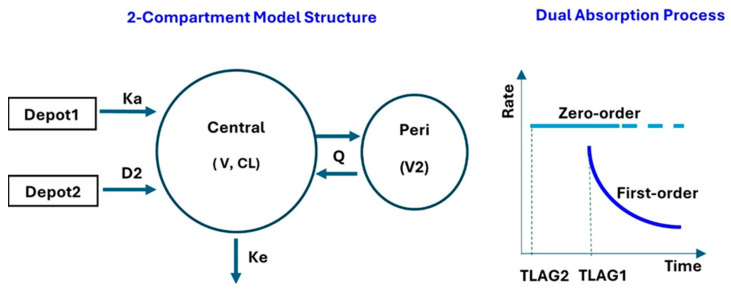
PopPK model structure. Schematic diagram of vatiquinone PopPK model structure (**left**) and dual parallel zero- and first-order absorption processes (**right**). CL: systemic clearance; D2: duration of absorption; Ka: absorption constant; Ke: first-order elimination rate constant; Peri: peripheral; PopPK: population pharmacokinetic; Q: compartmental transfer constant; TLAG1: lag time for first-order absorption process; TLAG2: lag time for zero-order absorption process; V: volume of distribution; V2: peripheral compartment volume of distribution.

Various combinations of compartment models (e.g., one-, two-, and three-compartment models) and absorption processes (e.g., sole absorption process, transit compartments, and dual absorption process) were explored. A two-compartment model with a parallel zero- and first-order absorption process following oral administration and first-order elimination was selected because it was identified as a robust model to describe the observations adequately. The model structure is depicted in Figure 5.

During the model development, a complete battery of diagnostic plots was generated to evaluate the adequacy of the model fit [25]. Plots of weighted residuals (WRES), individual weighted residuals (IWRES), and conditional weighted residuals (CWRES) versus time and predicted concentrations were evaluated for random scatter around the zero line. The CWRES plots were also used to identify potential outliers described as |CWRES| > 5.

### 4.7. Covariate Assessment

The relationship between continuous covariates and the typical value of PK parameters was described using power models:

$$\theta_{TV,ij}=\theta_{REF}{\left(\frac{X_{ij}}{X_{REF}}\right)}^{\theta_{x}}$$

where θ_REF_ and θ_x_ are the fixed-effect parameters, x_REF_ is a reference value of the covariate x, and x_ij_ is the value of covariate x for participant i at time j. For this analysis, either the median value in the population or a standard reference value (e.g., 65 kg) was used for x_REF_.

The relationship between categorical covariates (x_ij_) and the typical value of PK parameters was modelled as follows:

$$\theta_{TV,ij}=\theta_{REF}\cdot (1+\theta_{x}\cdot X_{ij})\;\mathrm{or}\;\theta_{TV,ij}=\theta_{REF}\cdot e^{\theta_{x}\cdot X_{ij}}$$

where θ_REF_ and θ_x_ are fixed-effect parameters and x_ij_ is the indicator variable which is equal to 1 or 0 dependent on the category of the covariates for participant i at time j.

A stepwise process, including forward addition and backward elimination procedures, was used to identify significant clinically relevant covariates for a parsimonious PK model. In forward addition, one covariate was evaluated each time and the covariate meeting the inclusion criterion of a change in the objective function value (ΔOFV) > 3.84 (*p* < 0.05, df = 1) was incorporated into the working full model. Then, in each round of backward elimination, the covariate–parameter relationship that had the lowest ΔOFV and did not meet the inclusion criterion of ΔOFV > 10.84 (*p* < 0.001, df = 1) was eliminated from the working full model and the procedure was repeated until all the covariate parameters in the final model satisfied the inclusion criteria.

### 4.8. Final Model Evaluation, Illustration of Covariate Effects, and Internal Validation

A visual predictive check (VPC) [26,27,28,29] was performed on the final model. Parameters were fixed to the final model estimates to simulate 1000 datasets that replicated the design, dose regimen, sample sizes, and covariate distributions in the observed dataset. The observed 5th, 50th, and 95th percentiles of vatiquinone concentrations were binned by time and compared visually to the 5th and 95th percentiles (90% confidence interval) of the 1000 simulated summary measures at the corresponding percentiles (5th, 50th, and 95th) of the simulated data to provide a visual assessment of the predictive performance of the PopPK model.

The impact illustration of predictive covariates from the final model on vatiquinone exposure was presented using forest plots. One thousand sets of population parameter values were generated using a smoothed parametric bootstrap procedure from the parameter matrix of the final model. Following oral administration of 400 mg of vatiquinone three times a day (TID), the maximum concentration at steady state (C_max,ss_), minimum concentration at steady state (Cmin,ss), and area under the concentration–time curve from 0 to 24 h at steady state (AUC_0–24h,ss_) were calculated for each covariate and presented in tabular format as well as in forest plots [30].

For internal validation, 1000 datasets each containing 100 patients with FA were sampled from clinical studies PTC743-NEU-003-FA and PTC743-NEU-005-FA with replacements using a posterior predictive check approach [31]. For each simulation, the observed and predicted PK concentrations, prediction error, absolute prediction error (APE), relative root mean square error (RMSE), mean prediction error (MPE), and mean absolute prediction error (MAPE) were calculated. The predictability of the model was satisfactory and clinically acceptable when the model met the pre-specified criteria of −10% ≤ PE ≤ 10%, APE < 20%, RMSE < 10%, −10% ≤ MPE ≤ 10%, and MAPE < 20% [10,11].

## 5. Conclusions

Based on the comprehensive PopPK modeling analysis, the concentration–time profiles of vatiquinone can be described using a two-compartment model with parallel zero- and first-order absorption processes and a linear elimination process. Compared to healthy volunteers, there was a decrease in CL and relative bioavailability (FK0) for patients with Friedreich’s ataxia (FA). This resulted in FA patients having decreases of 19% in C_max,ss_, 11% in C_min,ss_, and 24% in AUC_0–24h,ss_ in vatiquinone exposures at steady state. However, there were no noteworthy influences on PK exposures observed in patients with other mitochondrial diseases. Extrinsic factors such as meals, CYP3A4 inhibition by itraconazole and induction by rifampin, and intrinsic factors such as body weight and body mass index (BMI) significantly affected vatiquinone exposure after oral administration. Formulation, race, sex, age, pediatric maturation, and various hepatic and renal function biomarkers had no apparent impact on vatiquinone pharmacokinetic exposures.

## Figures and Tables

**Table 1:** Summary of participants and PK samples used in PopPK modeling analysis.

Study	Population	Participants, n ^a^	PK Samples, n ^b^
EPI743-12-001	Adult/HV	18	333
EPI743-18-002	Adult/HV	49	997
PTC743-NEU-004-FA	Adult/HV	16	466
PTC743-CNS-006-HV	Adult/HV	33	795
EPI-2010-006	Adult/FA	42	700
PTC743-NEU-003-FA	Adult/pediatric/FA	126	972
PTC743-NEU-005-FA	Pediatric/FA	5	34
PTC743-MIT-001-EP	Adult/pediatric/MD	54	311
Overall		343	4608

^a^ Participant numbers were accounted with at least one evaluable concentration data. ^b^ Data points from placebo group, BQL data, and all data points from participants with mishandled doses were excluded. Abbreviations: BQL: below the quantification limit; FA: Friedreich’s ataxia; HV: healthy volunteers; MD: mitochondrial disease; PK: pharmacokinetics; PopPK: population pharmacokinetics.

**Table 2:** Pharmacokinetic parameter estimates for the final model.

Theta/Parameter (Units)	Estimate	ASE		%RSE	90% CI	
1 FK0	0.744	0.021		2.823	0.710; 0.779	
2 K_a_ (1/h)	0.200	0.016		8.000	0.173; 0.228	
3 TK0 (hour)	6.034	0.102		1.690	5.866; 6.203	
4 TLAG1 (hour)	2.787	0.044		1.579	2.715; 2.859	
5 V/F (L)	180.748	21.38		11.829	145.471; 216.025	
6 CL/F (L/h)	162.721	10.14		6.232	145.990; 179.452	
7 V2/F (L)	4852.69	773.404		15.938	3576.573; 6128.807	
8 Q/F (L/h)	67.896	6.136		9.037	57.772; 78.019	
10 Itraconazole on CL/F	−1.446	0.175		−12.102	−1.735; −1.158	
11 Rifampin on CL/F	0.704	0.099		14.063	0.541; 0.867	
12 Liquid PediaSure^®^ on FK0	−2.671	0.130		−4.867	−2.886; −2.457	
13 Fasted statuses on FK0	−3.324	0.167		−5.024	−3.599; −3.050	
14 BWT on CL/F	0.915	0.123		13.443	0.712; 1.118	
15 Disease FA on CL/F	−0.406	0.082		−20.197	−0.541; −0.272	
16 Disease FA on FK0	−0.501	0.057		−11.377	−0.595; −0.408	
17 BMI on CL/F	−0.975	0.249		−25.538	−1.386; −0.564	
**Residual Variability**	**Estimate**	**ASE**		**%RSE**	**90% CI**	
9 Additive residual	1.062	0.011		1.036	1.043; 1.081	
**IIV**	**Estimate**	**ASE**		**(%CV)**	**(Shrinkage)**	
IIV–CL/F	0.191	0.025		45.880	24.370	
**OFV**	5488.13		**Condition number**			57.856

%CV: approximate coefficient of variation; %RSE: percent relative standard error; ASE: asymptotic standard error; BMI: body mass index; BWT: baseline body weight; CI: confidence interval; CL/F: apparent clearance; FA: Friedreich’s ataxia; FK0: absorption fraction via first-order absorption process; IIV: inter-individual variability; K_a_: absorption constant; OFV: objective function value; Q/F: apparent intercompartmental clearance; TK0: absorption duration, D2; TLAG1: delay time for first-order absorption process; V/F: apparent volume of the central compartment; V2/F: apparent volume of the peripheral compartment.

**Table 3:** Influence of covariates on vatiquinone exposure at steady state.

Covariate		Reference Population	Ratio Relative to Reference Mean Value (90% CI)			Clinical Relevance ^d^
			C_max,ss_	C_min,ss_	AUC_0–24h,ss_	
Reference		HV	1 (1; 1)	1 (1; 1)	1 (1; 1)	--
Disease FA on exposure ^a^		HV	0.76 (0.68; 0.85)	0.89 (0.78; 1.03)	0.81 (0.73; 0.91)	Most likely
Disease FA on FK0		HV	0.50 (0.4; 0.59)	0.50 (0.40; 0.59)	0.50 (0.4; 0.59)	Yes
Disease FA on CL/F		HV	1.54 (1.29; 1.91)	1.80 (1.42; 2.41)	1.65 (1.34; 2.1)	Yes
BMI ^b^	(5th, 13.82 kg/m^2^)	21.6 kg/m^2^	0.70 (0.60; 0.81)	0.60 (0.49; 0.75)	0.65 (0.55; 0.78)	Yes
	(95th, 30.67 kg/m^2^)	21.6 kg/m^2^	1.33 (1.18; 1.49)	1.47 (1.25; 1.72)	1.38 (1.21; 1.58)	Most likely
BWT ^b^	(5th, 17.14 kg)	65 kg	2.88 (2.35; 3.53)	3.16 (2.42; 4.13)	2.99 (2.37; 3.76)	Yes
	(95th, 96.66 kg)	65 kg	0.71 (0.66; 0.76)	0.70 (0.63; 0.77)	0.70 (0.65; 0.76)	Yes
Itraconazole (CYP3A4 inhibitor)		Vatiquinone monotherapy	3.04 (2.51; 3.61)	4.15 (3.31; 5.11)	3.52 (2.87; 4.27)	Yes
Rifampin (CYP3A4 inducer)		Vatiquinone monotherapy	0.55 (0.48; 0.63)	0.44 (0.37; 0.52)	0.50 (0.43; 0.58)	Yes
Liquid PediaSure^®^ meal		Medium-fat meal ^c^	0.07 (0.06; 0.09)	0.07 (0.06; 0.09)	0.07 (0.06; 0.09)	Yes
Fasted status		Medium-fat meal ^c^	0.04 (0.03; 0.05)	0.04 (0.03; 0.05)	0.04 (0.03; 0.05)	Yes

^a^ Overall disease FA’s effect on PK exposures. ^b^ The 5th and 95th percentiles vs. the reference value for participants in the analysis dataset. ^c^ A medium-fat meals have at least 25% calories from fat. ^d^ “Yes” denotes both the mean values and the 90% CI exceed the criteria values of 0.8–1.25 for all three PK parameters, while “Most likely” is defined as having at least one mean value exceed criteria, with the 90% CI boundary slightly overlapping the criteria values. Abbreviations: AUC_0–24h,ss_: area under the concentration–time curve from 0 to 24 h at steady state; BMI: baseline body mass index; BWT = baseline body weight; CI: confidence interval; CL/F: apparent clearance; C_max,ss_: maximum concentration at steady state; C_min,ss_: minimum concentration at steady state; FK0: fraction of absorption; FA: Friedreich’s ataxia; HV: healthy volunteers; PK: pharmacokinetics.

**Table 4:** Model performance evaluation parameters.

Parameter	Typical Prediction (PRED), Mean (90% CI)	Individual Prediction (IPRED), Mean (90% CI)	Prespecified Criteria ^a^
PE (%)	4.55 (−18.8, 38.0)	4.48 (−22.8, 39.1)	<±10%
APE (%)	11.90 (0.616, 38.4)	14.90 (0.901, 40.5)	<20%
RMSE (%)	0.84 (0.627, 1.06)	0.90 (0.711, 1.13)	<10%
MPE (%)	4.55 (1.59, 7.78)	4.48 (1.12, 8.00)	<±10%
MAPE (%)	11.90 (9.98, 14.2)	14.90 (12.7, 17.4)	<20%

^a^ Criteria were prespecified as a good-fit model. APE: absolute prediction error; CI: confidence interval; IPRED: individual prediction; MAPE: mean absolute prediction error; MPE: mean prediction error; PE: prediction error; PRED: typical prediction; RMSE: root mean square error.

**Table 5:** Tabular listing of studies used in PopPK modeling analysis.

Study	Population ^b^	Description	Dose Regimen	Plasma Sampling
EPI743-12-01 (NCT: NA)	HV, adults (n = 18)	Crossover, food effect study (fasted, liquid food PediaSure^®^, and medium-fat meals)	300 mg, single dose, capsule	Intensive (pre-dose, 0.5, 1, 1.5, 2, 3, 4, 6, 8, 12, 24, and 48 h post dose)
EPI743-18-002 (NCT: NA)	HV, adults (n = 49)	Crossover, DDI study with itraconazole or rifampin, medium-fat meals	400 mg, single dose, capsule	Intensive (Day 1 and 22: pre-dose, 1, 2, 3, 4, 5, 6, 8, 10, 12, 14, 16, 24, and 36 h post dose)
PTC743-NEU-004-FA ^a^ (NCT: NA)	HV, adults (n = 16)	Part 1: 7 days TID dosing, medium-fat meals Part 2: ^14^C, single dose	400 mg (n = 8), 200 mg (n = 8), multiple dose (TID), capsule	Intensive (Day 1 and 6: pre-dose, 2, 3, 4, 5, 6, 7, 8, 9, 10, 11, 12, 13, 14, 15, 24 h post morning dose)
PTC743-CNS-006-HV (NCT: NA)	HV, adults (n = 33)	Run-in phase, 4 treatments crossover TQT study, medium-fat meals	400 mg (n = 28), 1400 mg (n = 28), Placebo (n = 28), single dose, capsule	Intensive (Pre-dose, 1, 2, 3, 4, 5, 6, 7, 8, 10, 12, 14, 24, 36, and 48 h post dose)
EPI-2010-006 (NCT: 01728064)	FA patients, adults (n = 63)	Phase 2b 6-month safety/efficacy double-blind placebo controlled with 6-month extension, medium-fat meals	Placebo, 200 mg TID, 400 mg TID, multiple doses, capsule	Intensive (Day 1 and 3: pre-dose, 1, 2, 3, 4, 6, 8, 10, 11, 12 h post morning dose)
PTC743-NEU-003-FA (NCT: 04577352)	FA patients, pediatrics and adults, 8–67 years old (n = 146)	Randomized double-blind, placebo-controlled (72 weeks), medium-fat meals	200 mg (TID) if ˂12 years old and body weight ˂ 25 kg or a dose of 400 mg (TID) if ≥12 years old and/or body weight ≥ 25 kg or placebo (TID), multiple doses, capsule	Sparse (pre-dose at each visit of Week 1, 12, 24, 36, 48, 60, 72, 84, and 96)
PTC743-CNS-005-FA (NCT: 05485987)	Children with FA < 7 years old (n = 5)	An open-label, 72-week study to evaluate PK, safety, and efficacy of vatiquinone, medium-fat meals	15 mg/kg if body weight < 13 kg and 200 mg if body weight ≥ 13 kg, TID, multiple doses, solution	Sparse (Week 4, 12, and 24: pre-dose, 1 to 3 h, and 3–6 h post morning dose)
PTC743-MIT-001-EP (NCT: 04378075)	Mitochondria disease, pediatric patients < 19 years old (n = 94)	Randomized double-blind, placebo-controlled (24 weeks) Open-label extension (48 weeks), PediaSure meals	15 mg/kg if body weight < 13 kg, 200 mg if body weight ≥ 13 kg, multiple doses (TID), solution	Sparse (Day 1 and Week 24: pre-dose, 1, 3, 4, and 8 h post first dose; Week 48 and 72: 4 h post first dose)

^a^ Only part I of PTC743-NEU-004-FA was included in the PopPK analysis dataset. ^b^ n = planned enrollment number. DDI: drug–drug interaction study; FA: Friedreich’s ataxia; HV: healthy volunteers; a medium-fat meal = approximately 25% to 50% fat; NCT: National Clinical Trial Number; PK: pharmacokinetics; PopPK, population pharmacokinetics; TID: three times a day; TQT: thorough QT.

## Data Availability

A NONMEM format analysis dataset was generated based on raw PK data collected from clinical studies and is available upon reasonable request.

## Supplementary Materials

The following supporting information can be downloaded at https://www.mdpi.com/article/10.3390/ph18091339/s1, Figure S1: Observed vatiquinone concentration distribution (A) and concentration–time profile (B) overall and by study; Figure S2: The linearity analysis of vatiquinone PK exposure across doses; Figure S3: Selected vatiquinone concentration–time profiles; Figure S4: Goodness-of-fit plots of the final PopPK model; Figure S5: Selected VPC plots for evaluation of the final PopPK model; Table S1: Summary of categorical covariates in the final dataset for PK modelling analysis; Table S2: Summary of continuous covariates at baseline in the dataset for PK modelling analysis; Table S3: Assessment of dose linearity following a single dose (C_max_ and AUC_0–24h_); Table S4: Proportion of PK observations contained in 95% PI of predictions in an internal validation study stratified by body weight in FA patients; Table S5: Proportion of PK observations contained in 95% PI of predictions by population and body weight band; Table S6: Proportion of PK observations contained in 95% PI of predictions by population and age group; Table S7: Proportion of PK observations contained in 95% PI of predictions by study.
