# Supplementary material for: Characterizing Population Pharmacokinetics of Vatiquinone in Healthy Volunteers and Patients with Friedreich’s Ataxia
Source: Pharmaceuticals (Basel). 2025 Sep 6;18(9):1339. doi: 10.3390/ph18091339 (PMC12472293; doi:10.3390/ph18091339)
Supplement: Supplementary file 1 [file pharmaceuticals-18-01339-s001.zip › Supplementary of Pharmaceuticals-3803576.pdf]

Supplemental materials for Pharmaceuticals-3803576

**Supplementary Figure S1.** Observed vatiquinone concentration distribution (**A**) and concentration–time profile (**B**) overall and by study

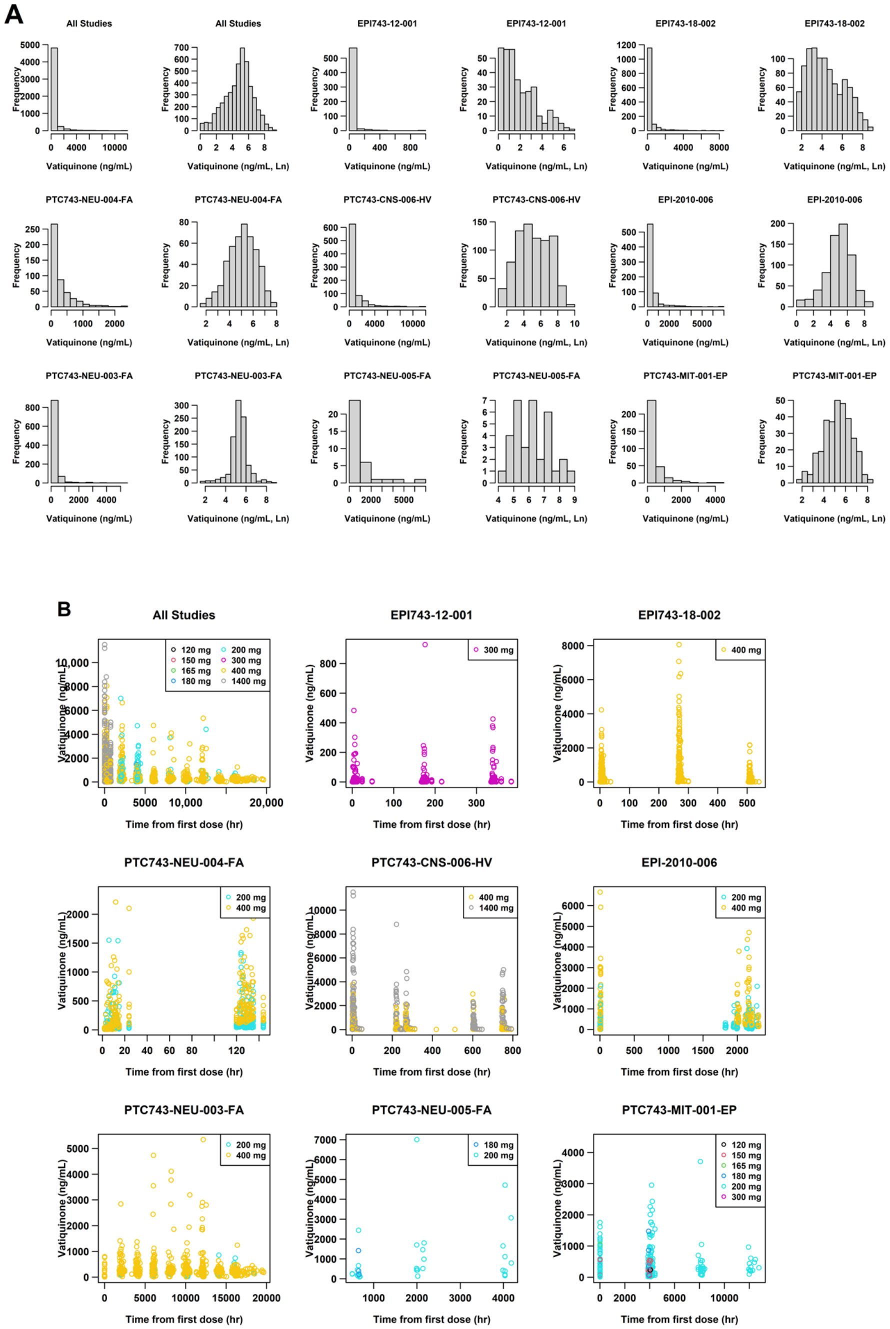


The circle indicates the pharmacokinetic observation for each time point.

**Supplementary Figure S2.** The linearity analysis of vatiquinone PK exposure across doses

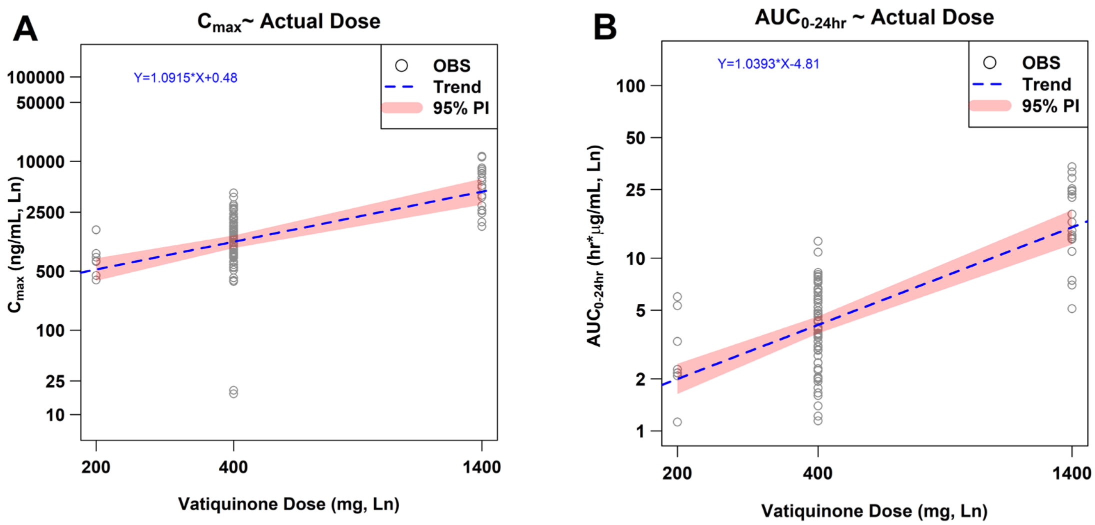


Dose proportionality is shown for $C_{max}$ (**A**) and $AUC_{0\text{-}24hr}$ (**B**). OBS indicates an observation (grey open circle) after administration of a single dose of vatiquinone, the blue dashed line indicates the central trend of PK exposure, and the pink shadow indicates the 95% PI after linear regression.

$AUC_{0\text{-}24hr}$: area under the concentration–time curve from 0 to 24 hours; $C_{max}$: maximum concentration; OBS: observed value; PI: prediction interval; PK: pharmacokinetics.

**Supplementary Figure S3.** Selected vatiquinone concentration–time profiles

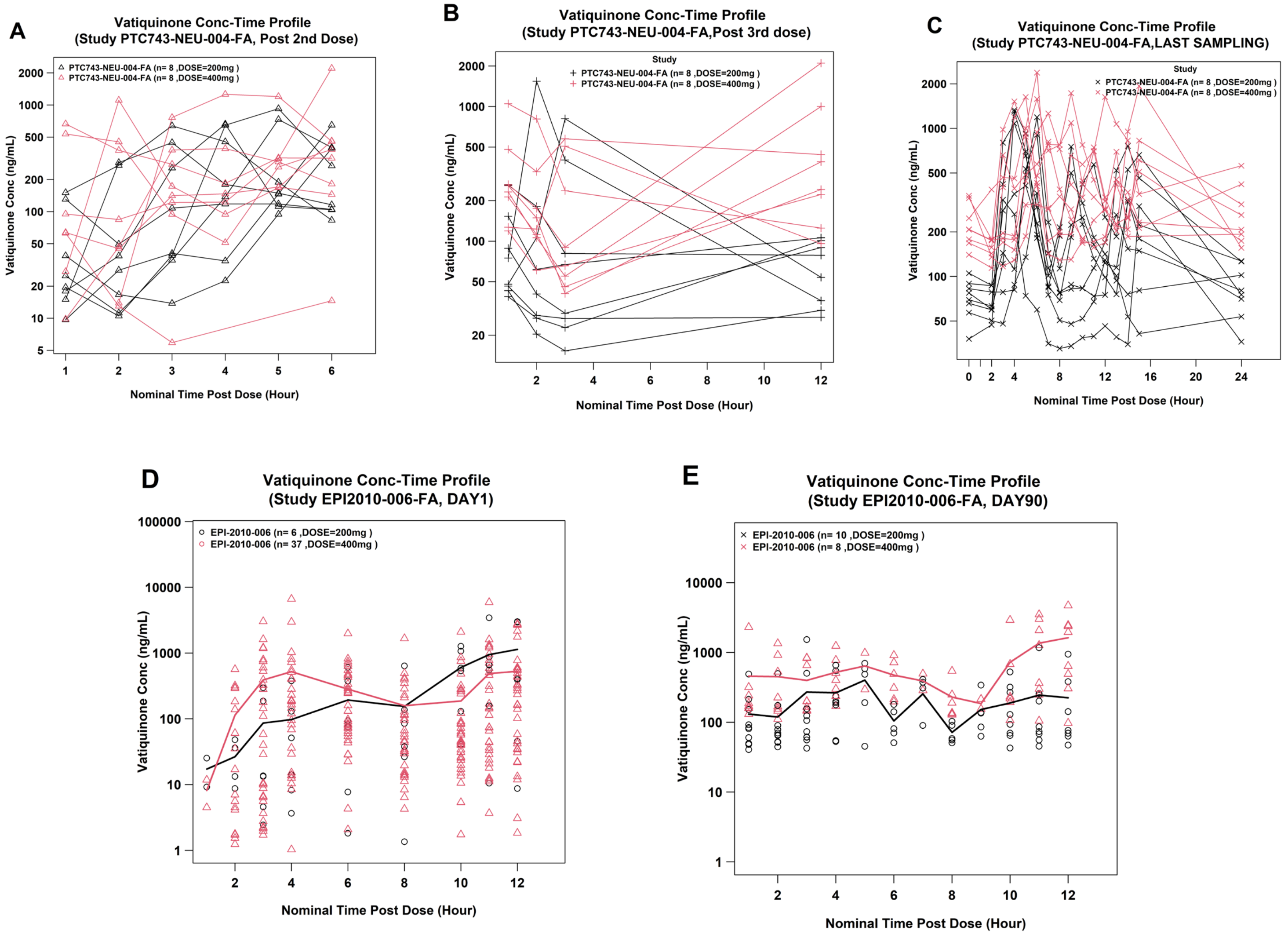


A notable delay in absorption phase, spanning 2-3 hours, was observed, as depicted in the plots exhibiting the lowest drug concentration at 2-3 hours after administration of the last dose in study PTC743-NEU-004-FA (**A**, **B** and **C**) and study EPI2010-006-FA (**D** and **E**).

**Supplementary Figure S4.** Goodness-of-fit plots of the final PopPK model

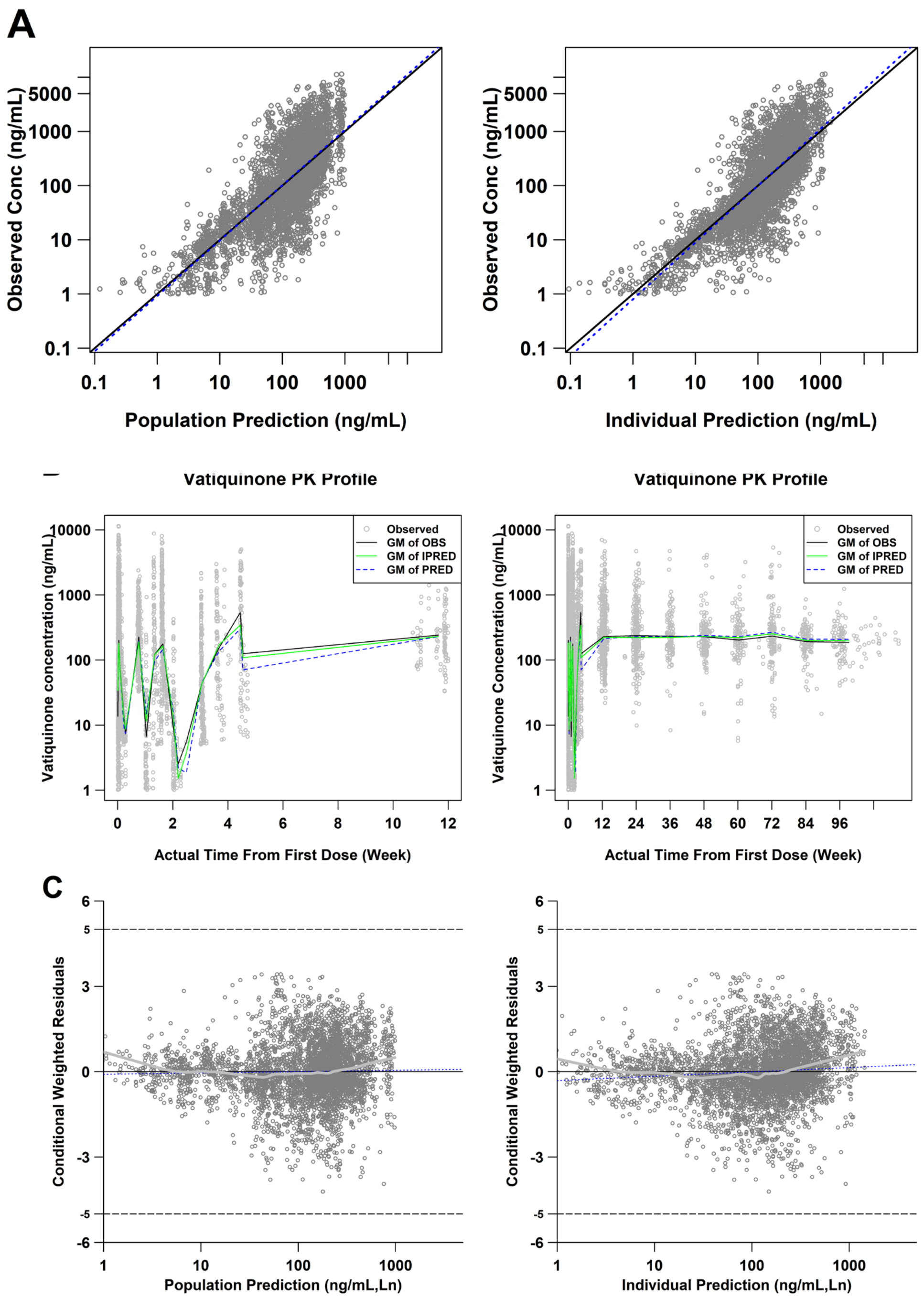

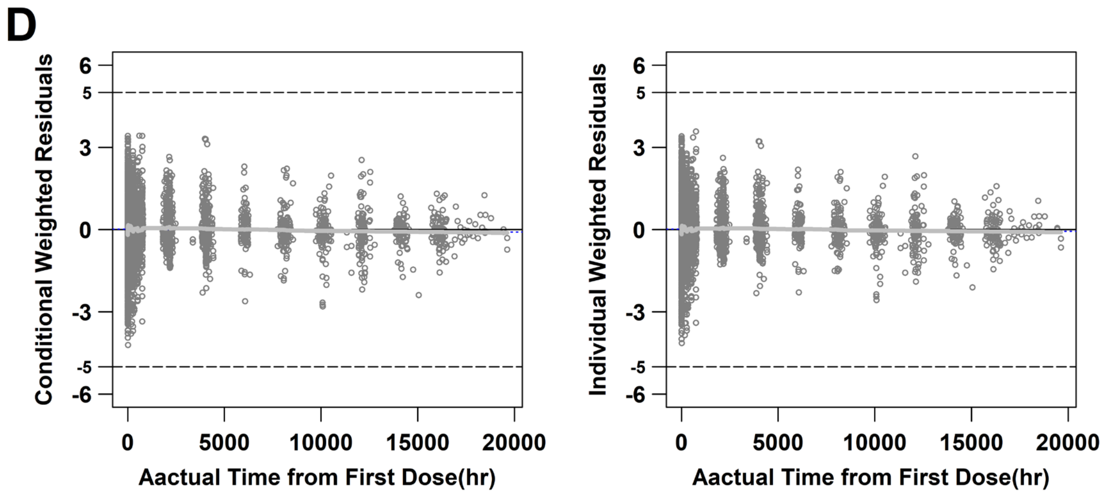


Population prediction and individual prediction versus observations of vatiquinone (**A**); overlaid profiles of the mean values of population prediction, individual prediction, and observation up to week 8 and overall (**B**); CWRES versus population prediction and individual prediction (**C**); and CWRES and IWRES versus time (**D**). CWRES: conditional weighted residuals; IPRED: individual prediction; IWRES: individual weighted residuals; OBS: observed value; PK: pharmacokinetics; PopPK: population pharmacokinetics; PRED: typical prediction.

**Supplementary Figure S5.** Selected VPC plots for evaluation of the final PopPK model

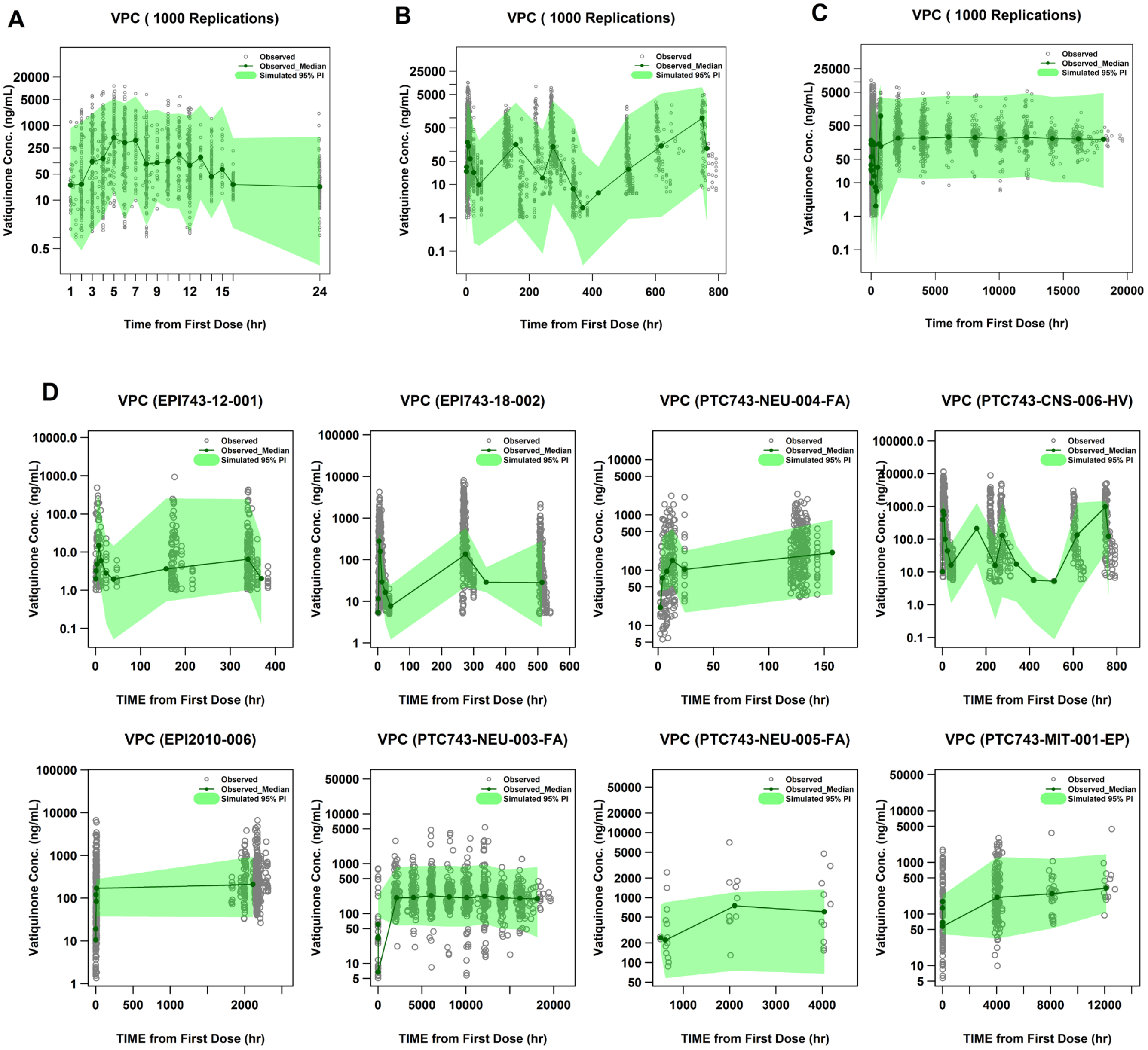


VPC plots with 95% prediction interval for up to 24 hours post dose (**A**), up to week 8 post dose (**B**), overall (**C**), and by individual study (**D**).

PopPK: population pharmacokinetics; VPC: visual predictive check

**Supplementary Table S1.** Summary of categorical covariates in the final dataset for PK modelling analysis

| | EPI743-12-001 | EPI743-18-002 | PTC743-NEU-004-FA | PTC743-CNS-006-HV | EPI-2010-006 | PTC743-NEU-003-FA | PTC743-NEU-005-FA | PTC743-MIT-001-EP | Overall |
|---|---|---|---|---|---|---|---|---|---|
| **Participants, N (%)** | (N=18) | (N=49) | (N=16) | (N=33) | (N=42) | (N=126) | (N=5) | (N=54) | (N=343) |
| **Sex** | | | | | | | | | |
| Females | 9 (50.0%) | 25 (51.0%) | 8 (50.0%) | 12 (36.4%) | 21 (50.0%) | 77 (61.1%) | 2 (40.0%) | 24 (44.4%) | 178 (51.9%) |
| Males | 9 (50.0%) | 24 (49.0%) | 8 (50.0%) | 21 (63.6%) | 21 (50.0%) | 49 (38.9%) | 3 (60.0%) | 30 (55.6%) | 165 (48.1%) |
| **Subpopulation** | | | | | | | | | |
| Adults (≥18 yo) | 18 (100%) | 49 (100%) | 16 (100%) | 33 (100%) | 42 (100%) | 40 (31.7%) | 0 (0%) | 1 (1.9%) | 199 (58.0%) |
| Pediatrics (<18 yo) | 0 (0%) | 0 (0%) | 0 (0%) | 0 (0%) | 0 (0%) | 86 (68.3%) | 5 (100%) | 53 (98.1%) | 144 (42.0%) |
| **Disease** | | | | | | | | | |
| HV | 18 (100%) | 49 (100%) | 16 (100%) | 33 (100%) | 0 (0%) | 0 (0%) | 0 (0%) | 0 (0%) | 116 (33.8%) |
| FA | 0 (0%) | 0 (0%) | 0 (0%) | 0 (0%) | 42 (100%) | 126 (100%) | 5 (100%) | 0 (0%) | 173 (50.4%) |
| MD | 0 (0%) | 0 (0%) | 0 (0%) | 0 (0%) | 0 (0%) | 0 (0%) | 0 (0%) | 54 (100%) | 54 (15.7%) |
| **Race** | | | | | | | | | |
| Black or African American | 2 (11.1%) | 6 (12.2%) | 7 (43.8%) | 18 (54.5%) | 0 (0%) | 0 (0%) | 0 (0%) | 2 (3.7%) | 35 (10.2%) |
| White | 16 (88.9%) | 43 (87.8%) | 6 (37.5%) | 13 (39.4%) | 42 (100%) | 118 (93.7%) | 5 (100%) | 36 (66.7%) | 279 (81.3%) |
| Asian | 0 (0%) | 0 (0%) | 2 (12.5%) | 1 (3.0%) | 0 (0%) | 0 (0%) | 0 (0%) | 9 (16.7%) | 12 (3.5%) |
| Missing | 0 (0%) | 0 (0%) | 1 (6.3%) | 1 (3.0%) | 0 (0%) | 8 (6.3%) | 0 (0%) | 7 (13.0%) | 17 (5.0%) |
| **Ethnicity** | | | | | | | | | |
| No | 18 (100%) | 1 (2.0%) | 12 (75.0%) | 29 (87.9%) | 42 (100%) | 108 (85.7%) | 5 (100%) | 52 (96.3%) | 267 (77.8%) |

| | EPI743-12-001 | EPI743-18-002 | PTC743-NEU-004-FA | PTC743-CNS-006-HV | EPI-2010-006 | PTC743-NEU-003-FA | PTC743-NEU-005-FA | PTC743-MIT-001-EP | Overall |
|---|---|---|---|---|---|---|---|---|---|
| **Participants, N (%)** | (N=18) | (N=49) | (N=16) | (N=33) | (N=42) | (N=126) | (N=5) | (N=54) | (N=343) |
| Yes | 0 (0%) | 48 (98.0%) | 4 (25.0%) | 4 (12.1%) | 0 (0%) | 18 (14.3%) | 0 (0%) | 2 (3.7%) | 76 (22.2%) |
| **Formulation** | | | | | | | | | |
| Capsule | 18 (100%) | 49 (100%) | 16 (100%) | 33 (100%) | 42 (100%) | 126 (100%) | 0 (0%) | 0 (0%) | 284 (82.8%) |
| Solution | 0 (0%) | 0 (0%) | 0 (0%) | 0 (0%) | 0 (0%) | 0 (0%) | 5 (100%) | 54 (100%) | 59 (17.2%) |
| **Food status** | | | | | | | | | |
| Fasted | 6 (33.3%) | 0 (0%) | 0 (0%) | 0 (0%) | 0 (0%) | 0 (0%) | 0 (0%) | 0 (0%) | 6 (1.7%) |
| Liquid | 6 (33.3%) | 0 (0%) | 0 (0%) | 0 (0%) | 0 (0%) | 0 (0%) | 0 (0%) | 0 (0%) | 6 (1.7%) |
| Medium fat | 6 (33.3%) | 49 (100%) | 16 (100%) | 33 (100%) | 42 (100%) | 126 (100%) | 5 (100%) | 54 (100%) | 331 (96.5%) |
| **Dose (mg)** | | | | | | | | | |
| 120 | 0 (0%) | 0 (0%) | 0 (0%) | 0 (0%) | 0 (0%) | 0 (0%) | 0 (0%) | 1 (1.9%) | 1 (0.3%) |
| 150 | 0 (0%) | 0 (0%) | 0 (0%) | 0 (0%) | 0 (0%) | 0 (0%) | 0 (0%) | 3 (5.6%) | 3 (0.9%) |
| 165 | 0 (0%) | 0 (0%) | 0 (0%) | 0 (0%) | 0 (0%) | 0 (0%) | 0 (0%) | 1 (1.9%) | 1 (0.3%) |
| 180 | 0 (0%) | 0 (0%) | 0 (0%) | 0 (0%) | 0 (0%) | 0 (0%) | 1 (20.0%) | 2 (3.7%) | 3 (0.9%) |
| 200 | 0 (0%) | 0 (0%) | 8 (50.0%) | 0 (0%) | 22 (52.4%) | 5 (4.0%) | 4 (80.0%) | 47 (87.0%) | 86 (25.1%) |
| 300 | 18 (100%) | 0 (0%) | 0 (0%) | 0 (0%) | 0 (0%) | 0 (0%) | 0 (0%) | 0 (0%) | 18 (5.2%) |
| 400 | 0 (0%) | 49 (100%) | 8 (50.0%) | 14 (42.4%) | 20 (47.6%) | 121 (96.0%) | 0 (0%) | 0 (0%) | 212 (61.8%) |
| 1400 | 0 (0%) | 0 (0%) | 0 (0%) | 19 (57.6%) | 0 (0%) | 0 (0%) | 0 (0%) | 0 (0%) | 19 (5.5%) |

A medium-fat meal is defined as food with at least 25% of calories from fat.

FA: Friedreich's ataxia; HV: healthy volunteers; MD: mitochondrial disease; PK: pharmacokinetics.

**Supplementary Table S2.** Summary of continuous covariates at baseline in the dataset for PK modelling analysis

| | EPI743-12-001 | EPI743-18-002 | PTC743-NEU-004-FA | PTC743-CNS-006-HV | EPI-2010-006 | PTC743-NEU-003-FA | PTC743-NEU-005-FA | PTC743-MIT-001-EP | Overall |
|---|---|---|---|---|---|---|---|---|---|
| **Participants (N)** | (N=18) | (N=49) | (N=16) | (N=33) | (N=42) | (N=126) | (N=5) | (N=54) | (N=343) |
| **Dose (mg)** | | | | | | | | | |
| N | 18 | 49 | 16 | 33 | 42 | 126 | 5 | 54 | 343 |
| Mean | 300 | 400 | 300 | 976 | 295 | 392 | 196 | 194 | 394 |
| SD | 0 | 0 | 103 | 502 | 101 | 39.2 | 8.94 | 16.4 | 260 |
| Median | 300 | 400 | 300 | 1400 | 200 | 400 | 200 | 200 | 400 |
| [Min, Max] | [300, 300] | [400, 400] | [200, 400] | [400, 1400] | [200, 400] | [200, 400] | [180, 200] | [120, 200] | [120, 1400] |
| **Age (years)** | | | | | | | | | |
| N | 18 | 49 | 16 | 33 | 42 | 126 | 5 | 54 | 343 |
| Mean | 34.9 | 34.5 | 37.1 | 43.1 | 28.9 | 18.1 | 5.20 | 7.83 | 24.1 |
| SD | 12.1 | 6.90 | 11.8 | 10.9 | 7.29 | 11.6 | 1.79 | 5.21 | 14.8 |
| Median | 33.5 | 35.0 | 35.5 | 40.0 | 27.0 | 15.0 | 6.00 | 7.00 | 20.0 |
| [Min, Max] | [18.0, 55.0] | [18.0, 45.0] | [21.0, 64.0] | [24.0, 63.0] | [18.0, 44.0] | [8.00, 67.0] | [2.00, 6.00] | [1.00, 19.0] | [1.00, 67.0] |
| **Body weight (kg)** | | | | | | | | | |
| N | 18 | 49 | 16 | 33 | 42 | 126 | 5 | 53 | 342 |
| Mean | 72.4 | 73.1 | 74.2 | 83.9 | 65.6 | 51.7 | 19.4 | 24.8 | 57.1 |
| SD | 14.3 | 10.5 | 12.7 | 13.6 | 16.6 | 17.6 | 4.65 | 15.0 | 23.8 |
| Median | 72.3 | 73.0 | 74.9 | 84.3 | 60.3 | 48.7 | 20.0 | 19.7 | 58.6 |
| [Min, Max] | [52.6, 106] | [57.5, 98.6] | [55.3, 99.8] | [55.2, 110] | [44.0, 112] | [21.2, 119] | [12.8, 25.8] | [6.30, 78.1] | [6.30, 119] |

| | EPI743-12-001 | EPI743-18-002 | PTC743-NEU-004-FA | PTC743-CNS-006-HV | EPI-2010-006 | PTC743-NEU-003-FA | PTC743-NEU-005-FA | PTC743-MIT-001-EP | Overall |
|---|---|---|---|---|---|---|---|---|---|
| **Participants (N)** | (N=18) | (N=49) | (N=16) | (N=33) | (N=42) | (N=126) | (N=5) | (N=54) | (N=343) |
| Missing | 0 (0%) | 0 (0%) | 0 (0%) | 0 (0%) | 0 (0%) | 0 (0%) | 0 (0%) | 1 (1.9%) | 1 (0.3%) |
| **BMI (kg/m$^2$)** | | | | | | | | | |
| N | 18 | 49 | 16 | 33 | 42 | 126 | 5 | 53 | 342 |
| Mean | 24.7 | 26.4 | 25.5 | 27.8 | 22.8 | 20.7 | 15.3 | 17.0 | 22.2 |
| SD | 4.70 | 2.32 | 3.08 | 2.67 | 4.69 | 5.47 | 1.50 | 3.64 | 5.55 |
| Median | 23.8 | 26.2 | 25.2 | 28.1 | 21.1 | 19.6 | 14.9 | 16.3 | 21.6 |
| [Min, Max] | [18.1, 32.0] | [19.8, 30.0] | [20.5, 30.2] | [20.1, 31.9] | [15.7, 36.4] | [12.5, 41.4] | [13.3, 17.3] | [11.9, 32.1] | [11.9, 41.4] |
| Missing | 0 (0%) | 0 (0%) | 0 (0%) | 0 (0%) | 0 (0%) | 0 (0%) | 0 (0%) | 1 (1.9%) | 1 (0.3%) |
| **Albumin (g/dL)** | | | | | | | | | |
| N | 0 | 49 | 16 | 33 | 42 | 126 | 5 | 54 | 325 |
| Mean | NA | 46.4 | 43.1 | 45.6 | 45.5 | 46.9 | 44.2 | 45.9 | 46.1 |
| SD | NA | 2.19 | 2.22 | 3.19 | 3.40 | 2.90 | 2.17 | 3.41 | 3.07 |
| Median | NA | 46.0 | 42.0 | 45.0 | 46.0 | 47.0 | 44.0 | 46.0 | 46.0 |
| [Min, Max] | NA | [41.0, 51.0] | [39.0, 48.0] | [40.0, 52.0] | [37.0, 51.0] | [39.0, 54.0] | [41.0, 47.0] | [38.0, 53.0] | [37.0, 54.0] |
| Missing | 18 (100%) | 0 (0%) | 0 (0%) | 0 (0%) | 0 (0%) | 0 (0%) | 0 (0%) | 0 (0%) | 18 (5.2%) |
| **Bilirubin (mg/dL)** | | | | | | | | | |
| N | 18 | 49 | 16 | 33 | 42 | 126 | 5 | 41 | 330 |
| Mean | 8.55 | 11.5 | 6.20 | 8.45 | 9.53 | 6.96 | 7.87 | 4.65 | 7.89 |
| SD | 3.57 | 3.42 | 2.99 | 6.03 | 4.12 | 4.50 | 1.53 | 1.86 | 4.55 |

| | EPI743-12-001 | EPI743-18-002 | PTC743-NEU-004-FA | PTC743-CNS-006-HV | EPI-2010-006 | PTC743-NEU-003-FA | PTC743-NEU-005-FA | PTC743-MIT-001-EP | Overall |
|---|---|---|---|---|---|---|---|---|---|
| **Participants (N)** | (N=18) | (N=49) | (N=16) | (N=33) | (N=42) | (N=126) | (N=5) | (N=54) | (N=343) |
| Median | 7.70 | 10.3 | 5.13 | 5.13 | 8.55 | 5.50 | 6.84 | 4.00 | 6.84 |
| [Min, Max] | [3.42, 17.1] | [6.84, 20.5] | [3.42, 12.0] | [3.42, 25.7] | [3.42, 20.5] | [2.60, 24.3] | [6.84, 10.3] | [2.00, 9.00] | [2.00, 25.7] |
| Missing | 0 (0%) | 0 (0%) | 0 (0%) | 0 (0%) | 0 (0%) | 0 (0%) | 0 (0%) | 13 (24.1%) | 13 (3.8%) |
| **AST (IU/L)** | | | | | | | | | |
| N | 18 | 49 | 16 | 33 | 42 | 126 | 5 | 54 | 343 |
| Mean | 17.5 | 20.2 | 18.3 | 20.8 | 26.7 | 22.1 | 43.0 | 23.1 | 22.3 |
| SD | 2.90 | 4.20 | 4.24 | 4.50 | 14.8 | 7.58 | 11.8 | 10.9 | 9.17 |
| Median | 17.0 | 19.0 | 17.5 | 21.0 | 22.0 | 20.5 | 41.0 | 20.0 | 20.0 |
| [Min, Max] | [13.0, 22.0] | [12.0, 29.0] | [14.0, 29.0] | [11.0, 31.0] | [14.0, 89.0] | [10.0, 55.0] | [29.0, 59.0] | [9.00, 61.0] | [9.00, 89.0] |
| **ALP (IU/L)** | | | | | | | | | |
| N | 18 | 49 | 16 | 33 | 42 | 126 | 5 | 54 | 343 |
| Mean | 88.2 | 65.4 | 68.0 | 71.6 | 67.2 | 142 | 177 | 180 | 115 |
| SD | 21.7 | 20.6 | 16.4 | 17.4 | 17.1 | 79.9 | 43.2 | 59.3 | 70.8 |
| Median | 91.0 | 60.0 | 65.0 | 71.0 | 64.0 | 128 | 168 | 178 | 85.0 |
| [Min, Max] | [50.0, 125] | [36.0, 132] | [45.0, 93.0] | [28.0, 103] | [43.0, 135] | [26.0, 332] | [135, 250] | [73.0, 352] | [26.0, 352] |
| **ALT (IU/L)** | | | | | | | | | |
| N | 18 | 49 | 16 | 33 | 42 | 126 | 5 | 51 | 340 |
| Mean | 37.3 | 23.1 | 15.8 | 19.2 | 27.3 | 17.4 | 23.0 | 18.6 | 20.8 |
| SD | 8.13 | 11.8 | 5.96 | 8.94 | 18.8 | 12.3 | 2.55 | 11.2 | 13.3 |

| | EPI743-12-001 | EPI743-18-002 | PTC743-NEU-004-FA | PTC743-CNS-006-HV | EPI-2010-006 | PTC743-NEU-003-FA | PTC743-NEU-005-FA | PTC743-MIT-001-EP | Overall |
|---|---|---|---|---|---|---|---|---|---|
| **Participants (N)** | (N=18) | (N=49) | (N=16) | (N=33) | (N=42) | (N=126) | (N=5) | (N=54) | (N=343) |
| Median | 34.0 | 19.0 | 17.0 | 18.0 | 24.5 | 14.0 | 23.0 | 17.0 | 17.0 |
| [Min, Max] | [29.0, 61.0] | [11.0, 59.0] | [7.00, 25.0] | [8.00, 49.0] | [10.0, 113] | [7.00, 127] | [20.0, 26.0] | [5.00, 67.0] | [5.00, 127] |
| Missing | 0 (0%) | 0 (0%) | 0 (0%) | 0 (0%) | 0 (0%) | 0 (0%) | 0 (0%) | 3 (5.6%) | 3 (0.9%) |
| **Creatinine (mg/dL)** | | | | | | | | | |
| N | 18 | 49 | 16 | 33 | 42 | 126 | 5 | 54 | 343 |
| Mean | 79.1 | 76.4 | 79.0 | 83.3 | 66.8 | 44.4 | 27.4 | 33.7 | 57.0 |
| SD | 15.0 | 13.2 | 16.0 | 14.3 | 13.0 | 13.3 | 7.83 | 10.6 | 22.5 |
| Median | 79.6 | 74.3 | 79.6 | 88.4 | 61.9 | 44.0 | 23.9 | 31.5 | 55.0 |
| [Min, Max] | [53.0, 106] | [52.2, 112] | [44.2, 115] | [44.2, 106] | [44.2, 90.2] | [27.0, 80.0] | [20.3, 37.1] | [18.0, 62.0] | [18.0, 115] |
| **CRCL (mL/min)** | | | | | | | | | |
| N | 18 | 49 | 16 | 33 | 42 | 126 | 5 | 53 | 342 |
| Mean | 110 | 117 | 111 | 116 | 126 | 164 | 118 | 111 | 134 |
| SD | 19.8 | 24.3 | 15.6 | 26.2 | 32.2 | 57.9 | 52.1 | 55.0 | 50.7 |
| Median | 102 | 111 | 111 | 112 | 119 | 153 | 102 | 98.0 | 123 |
| [Min, Max] | [85.0, 142] | [73.0, 203] | [86.0, 140] | [59.0, 183] | [79.0, 204] | [75.0, 472] | [81.0, 209] | [47.0, 345] | [47.0, 472] |
| Missing | 0 (0%) | 0 (0%) | 0 (0%) | 0 (0%) | 0 (0%) | 0 (0%) | 0 (0%) | 1 (1.9%) | 1 (0.3%) |

Creatinine clearance was calculated by: (140-AGE)*WEIGHT*1.23/(CREAT)*0.85 for females and (140-AGE)*WEIGHT*1.23/(CREAT) for males.

ALP: alkaline phosphatase; ALT: alanine aminotransferase; AST: aspartate aminotransferase; BMI: body mass index; CRCL: creatinine clearance; max: maximum; min: minimum; N: number of participants; PK: pharmacokinetics; SD: standard deviation.

**Supplementary Table S3.** Assessment of dose linearity following a single dose ($C_{max}$ and $AUC_{0\text{-}24hr}$)

| Parameter | Power coefficient estimate (γ)[a] | Standard error | 95% CI | Dose range (mg) | No. of participants | Beta 1 range based on literature[b] |
|---|---|---|---|---|---|---|
| $C_{max}$ | 1.09 | 0.15 | (0.80, 1.39) | 200-1400 | 98 | (0.64, 1.35) |
| $AUC_{0\text{-}24hr}$ | 1.04 | 0.097 | (0.85, 1.23) | 200-1400 | 98 | (0.64, 1.35) |

[a]The power estimate (γ) was obtained by linear regression of the log($C_{max}$) or log($AUC_{0\text{-}24hr}$) against the log(dose). [b]The beta 1 range was obtained based on the method outlined in reference [1].

$AUC_{0\text{-}24h}$: area under the concentration–time curve from 0 to 24 hours; CI: confidence interval; $C_{max}$: maximum concentration.

**Supplementary Table S4.** Proportion of PK observations contained in 95% PI of predictions in an internal validation study stratified by body weight in FA patients

| Body weight group | Visit time post dose, week | Participants, n | PK observations, n | Proportion of observed PK samples contained in 95% PI of prediction[a], % |
|---|---|---|---|---|
| <25 kg | 12 | 2 | 2 | 100 |
| | 24 | 2 | 2 | 100 |
| | 36 | 2 | 2 | 100 |
| | 48 | 1 | 1 | 100 |
| | 60 | 1 | 1 | 100 |
| | 84 | 1 | 1 | 100 |
| | 96 | 1 | 1 | 100 |
| | 108 | 1 | 1 | 100 |
| ≥25kg | 12 | 38 | 38 | 100 |
| | 24 | 24 | 24 | 100 |
| | 36 | 24 | 24 | 100 |
| | 48 | 27 | 27 | 100 |
| | 60 | 32 | 32 | 97 |
| | 72 | 29 | 29 | 93 |
| | 84 | 9 | 9 | 97 |
| | 96 | 12 | 12 | 100 |
| | 108 | 12 | 12 | 100 |

The 1000 datasets were generated by resampling from the PTC743-NEU-003-FA and PTC743-NEU-005-FA studies; each dataset includes 100 participants.

[a]1000 replicates in simulation.

FA: Friedreich's ataxia; PI: prediction interval; PK: pharmacokinetic.

**Supplementary Table S5.** Proportion of PK observations contained in 95% PI of predictions by population and body weight band

| Population | Body weight group | Participants, n[b] | Observed PK samples, n | Proportion of observed PK samples contained in 95% PI of prediction[a], % |
|---|---|---|---|---|
| Healthy volunteers | ≥25 kg | 116 | 2591 | 92.20 |
| FA patients | <13kg | 1 | 7 | 85.71 |
| | [13-25) kg | 8 | 42 | 92.86 |
| | ≥25 kg | 164 | 1657 | 93.36 |
| MD patients | <13kg | 10 | 45 | 93.33 |
| | [13-25) kg | 29 | 161 | 90.68 |
| | ≥25 kg | 15 | 105 | 93.33 |

[a]1000 replications in simulation. [b]Placebo excluded.

FA: Friedreich's ataxia; MD: mitochondrial disease; PI: prediction interval; PK, pharmacokinetics.

**Supplementary Table S6.** Proportion of PK observations contained in 95% PI of predictions by population and age group

| Population | Age group | Participants, n[b] | Observed PK samples, n | Proportion of observed PK samples contained in 95% PI of prediction[a], % |
|---|---|---|---|---|
| Healthy volunteers | >12 years old | 116 | 2591 | 92.20 |
| FA patients | <7 years old | 5 | 34 | 91.18 |
| | 7-12 years old | 32 | 200 | 97.50 |
| | >12 years old | 136 | 1472 | 92.80 |
| MD patients | <7 years old | 24 | 133 | 93.98 |
| | 7-12 years old | 17 | 92 | 86.96 |
| | >12 years od | 13 | 86 | 94.19 |

[a]1000 replications in simulation. [b]Placebo excluded.

FA: Friedreich's ataxia; MD: mitochondrial disease; PI=prediction interval; PK, pharmacokinetics.

**Supplementary Table S7.** Proportion of PK observations contained in 95% PI of predictions by study

| Study | Population | Participants,n [b] | Observed PK samples,n | Proportion of % observed PK samples contained in 95% PI of prediction[a],% |
|---|---|---|---|---|
| EPI743-12-01-FE | Healthy volunteers | 18 | 333 | 90.99 |
| EPI743-18-002-DDI | Healthy volunteers | 49 | 997 | 92.48 |
| PTC743-NEU-004-FA | Healthy volunteers | 16 | 466 | 95.49 |
| PTC743-CNS-006-HV | Healthy volunteers | 33 | 795 | 90.44 |
| EPI-2010-006 | Patients with FA | 42 | 700 | 88.29 |
| PTC743-NEU-003-FA | Patients with FA | 126 | 972 | 97.02 |
| PTC743-NEU-005-FA | Patients with FA | 5 | 34 | 91.18 |
| PTC743-MIT-001-EP | Patients with MD | 54 | 311 | 91.96 |

[a]1000 replications in simulation. [b]Placebo excluded.

FA: Friedreich's ataxia; MD: mitochondrial disease; PI=prediction interval; PK, pharmacokinetics.

# References

1. Hummel J, McKendrick S, Brindley C, French R (2009) Exploratory assessment of dose proportionality: review of current approaches and proposal for a practical criterion. Pharm Stat 8 (1):38-49. doi:10.1002/pst.326
