# Supplementary figures and images for: Characterizing Population Pharmacokinetics of Vatiquinone in Healthy Volunteers and Patients with Friedreich’s Ataxia
Source: Pharmaceuticals (Basel). 2025 Sep 6;18(9):1339. doi: 10.3390/ph18091339 (PMC12472293; doi:10.3390/ph18091339)

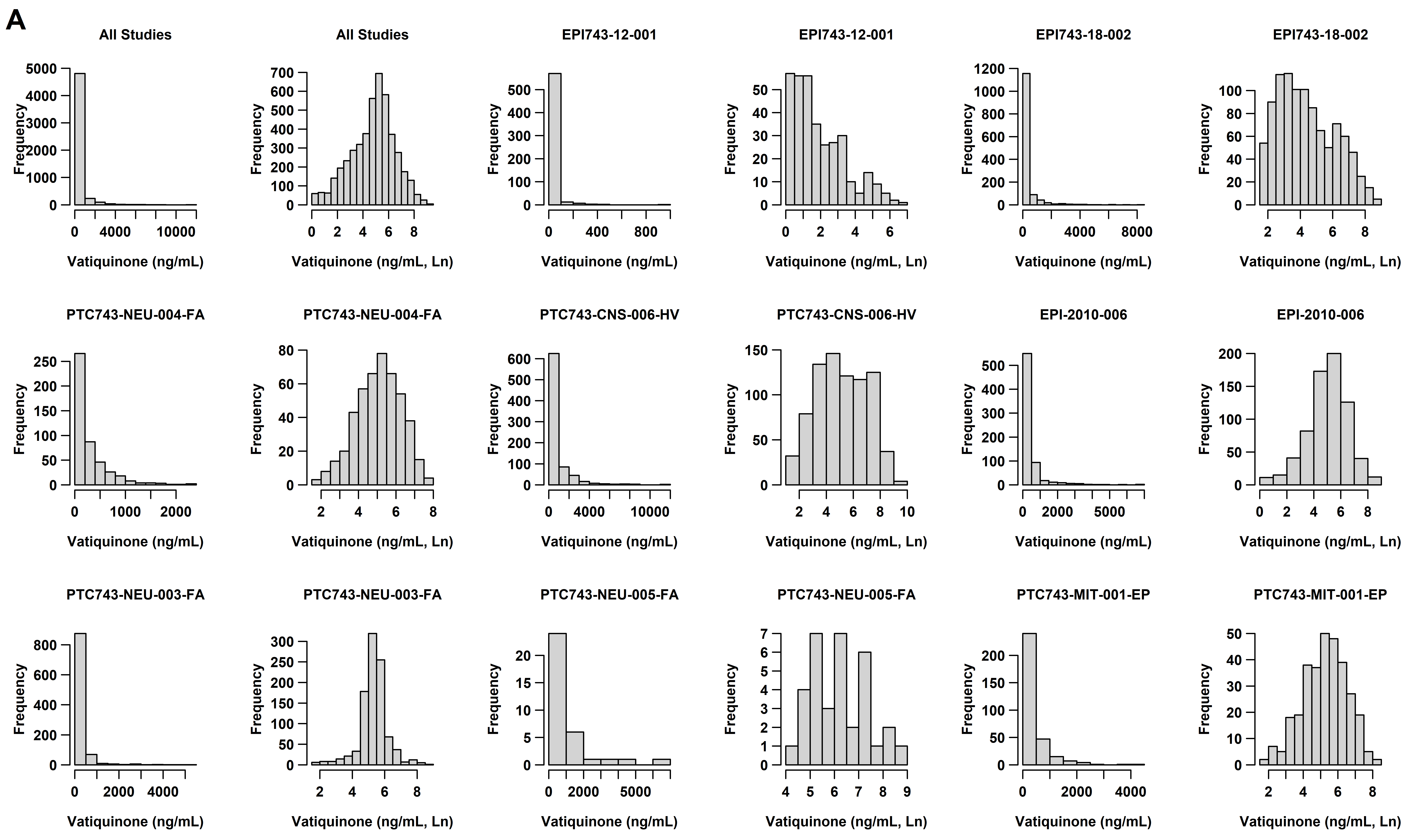

Supplement: Supplementary file 1 [file pharmaceuticals-18-01339-s001.zip › Figure S1A.png]

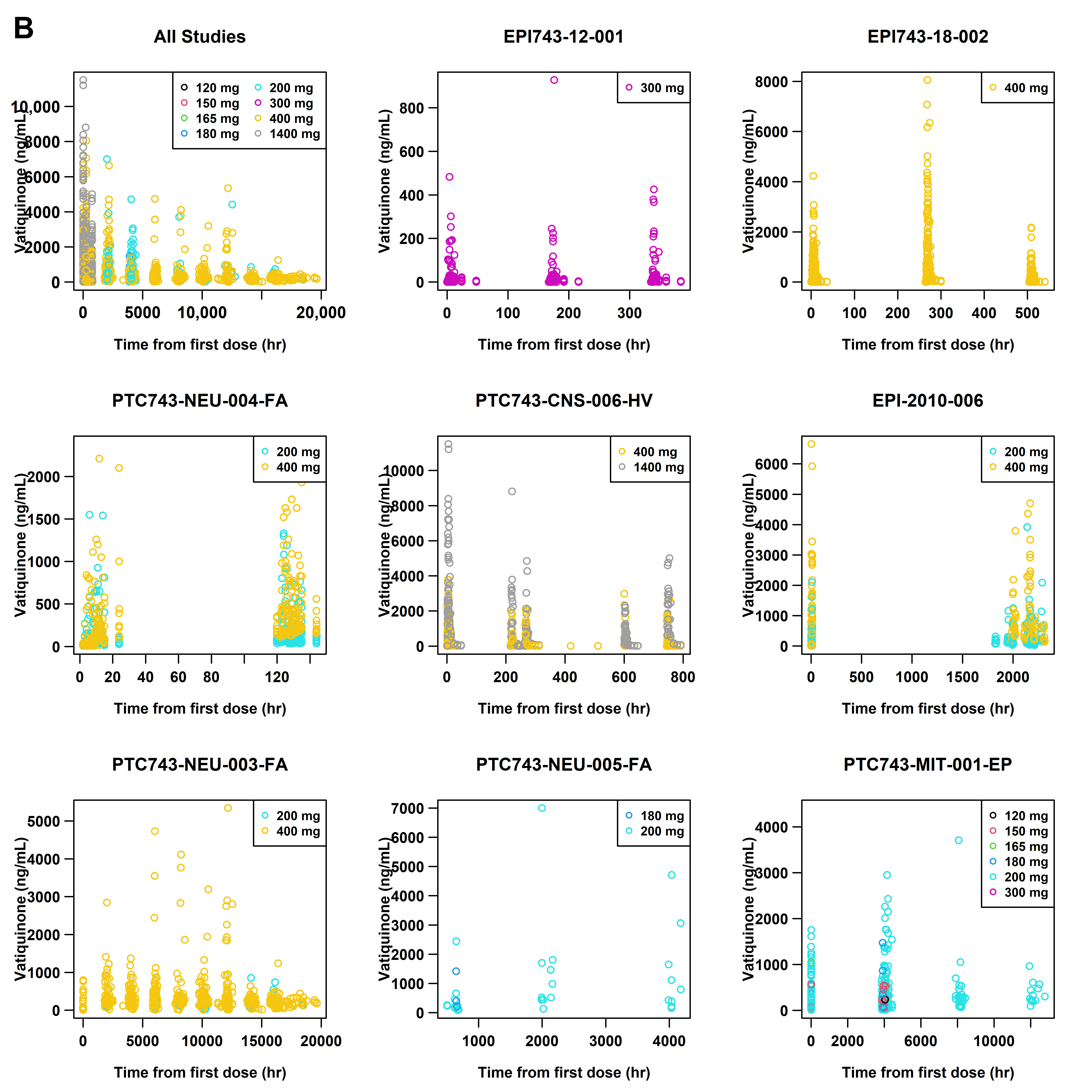

Supplement: Supplementary file 1 [file pharmaceuticals-18-01339-s001.zip › Figure S1B.png]

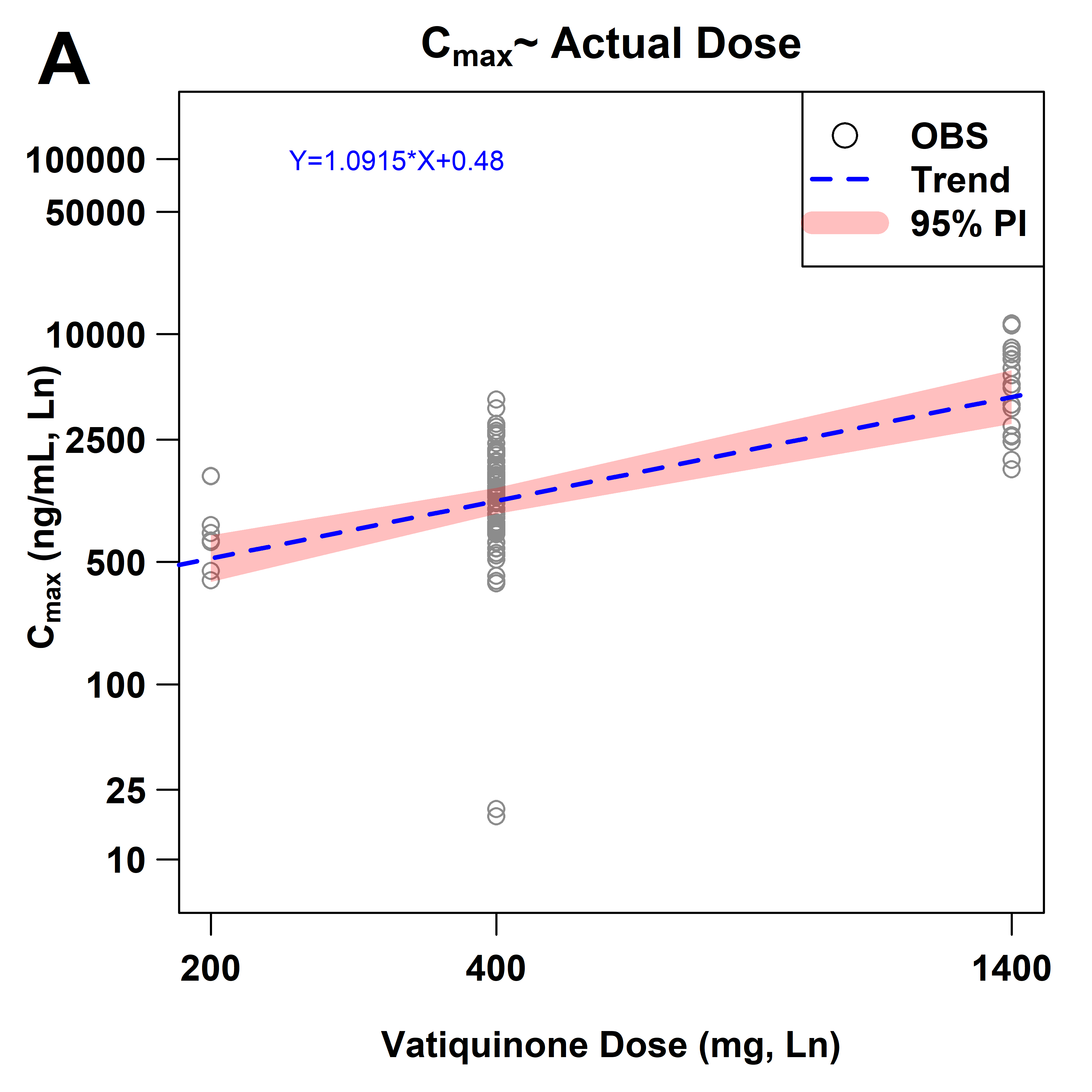

Supplement: Supplementary file 1 [file pharmaceuticals-18-01339-s001.zip › Figure S2A.png]

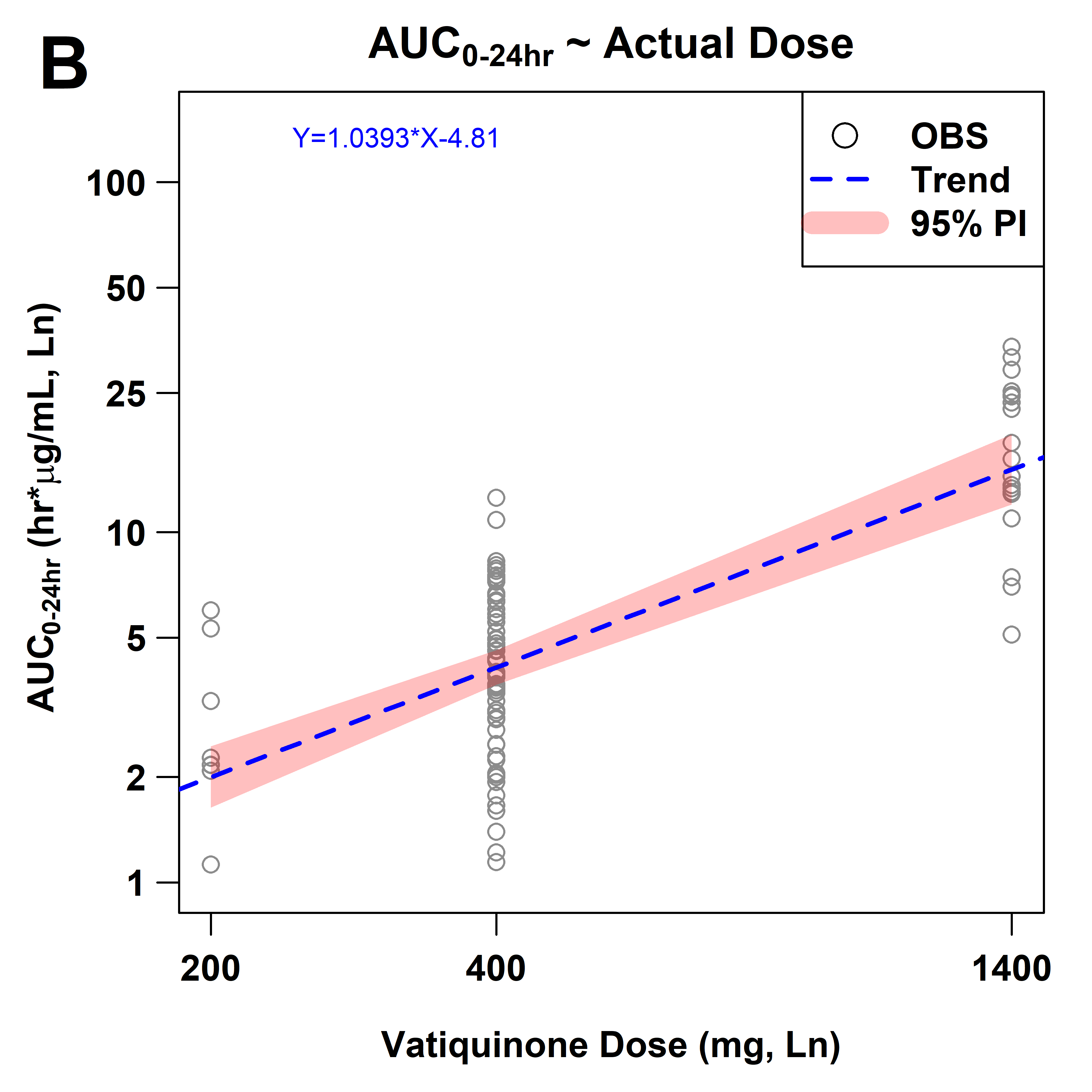

Supplement: Supplementary file 1 [file pharmaceuticals-18-01339-s001.zip › Figure S2B.png]

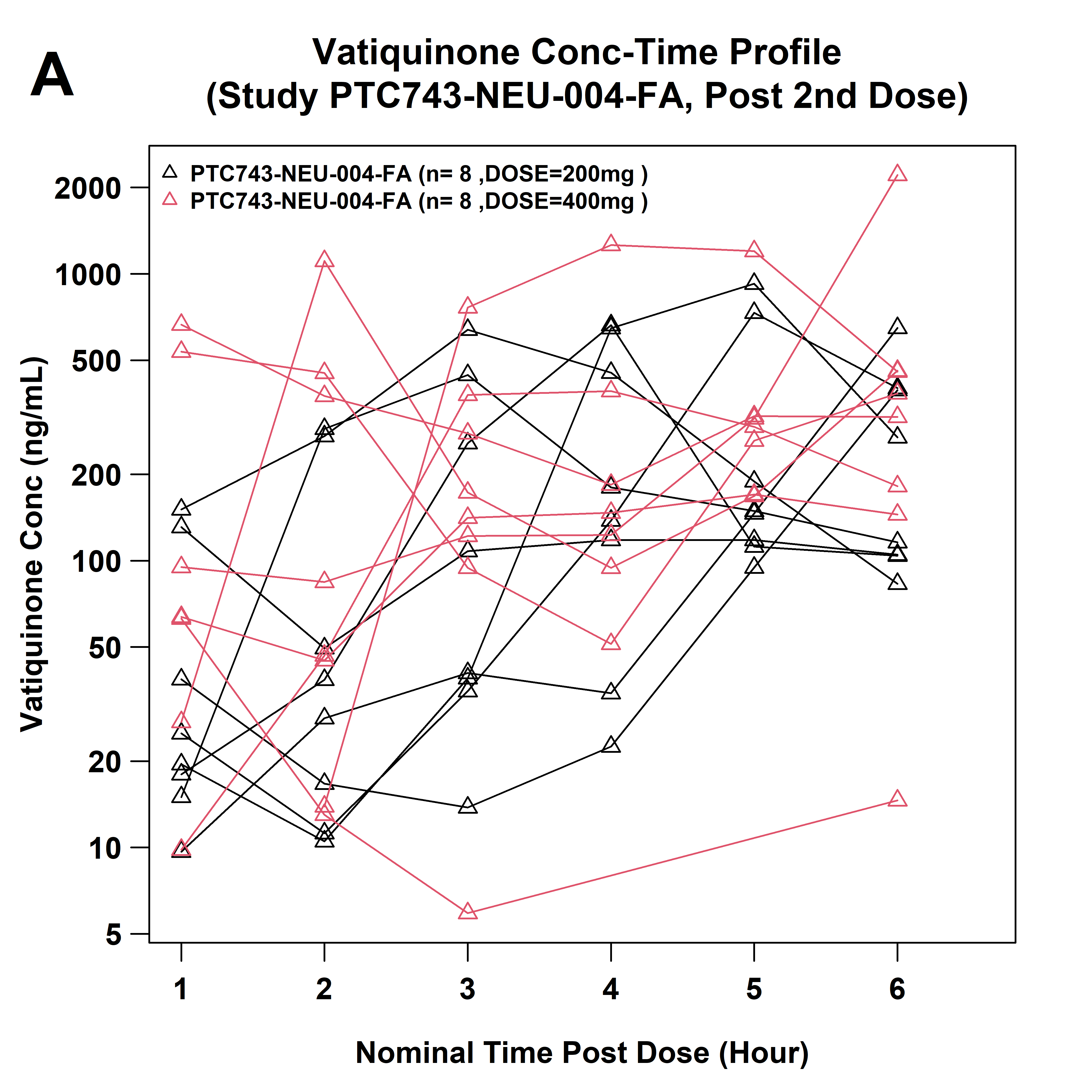

Supplement: Supplementary file 1 [file pharmaceuticals-18-01339-s001.zip › Figure S3A.png]

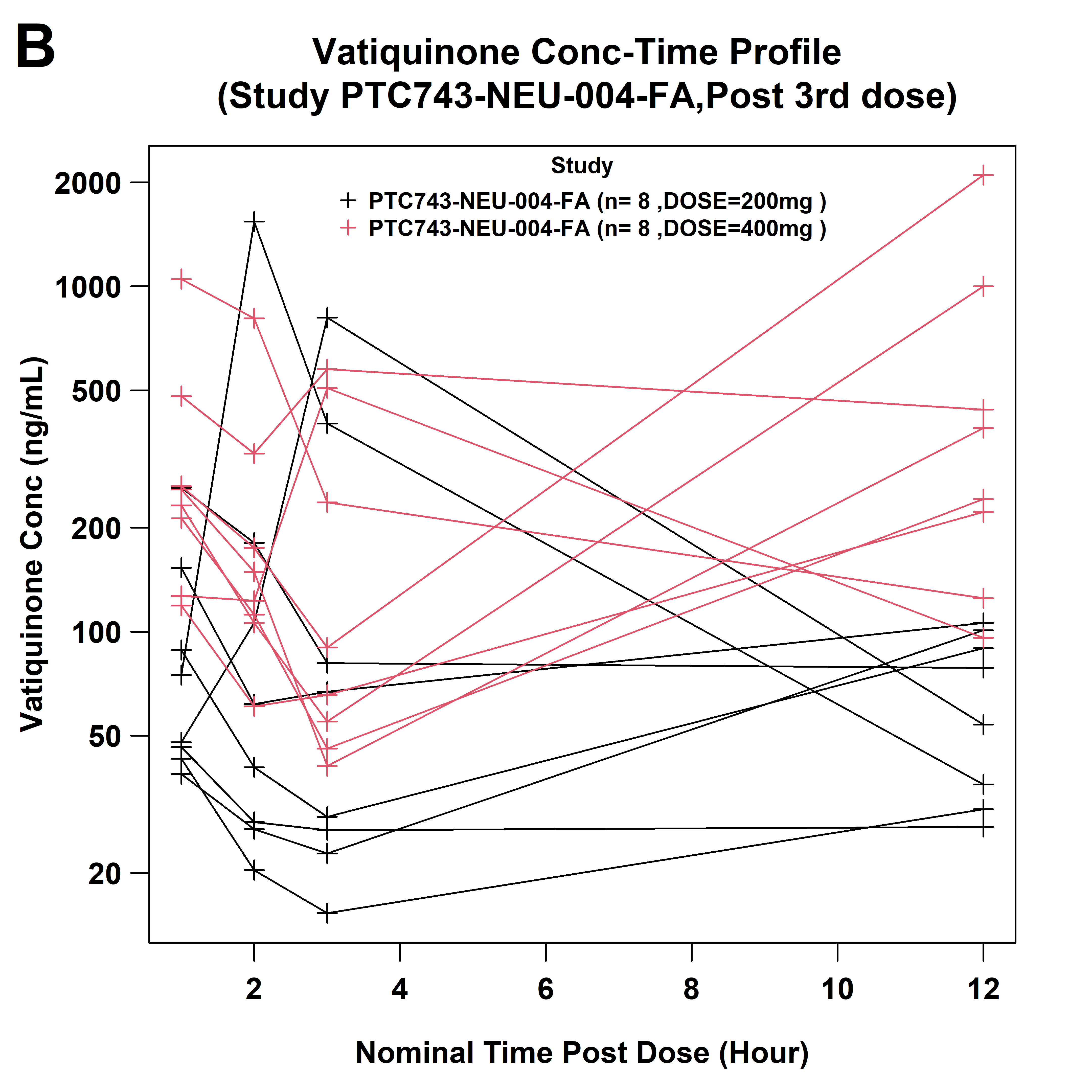

Supplement: Supplementary file 1 [file pharmaceuticals-18-01339-s001.zip › Figure S3B.png]

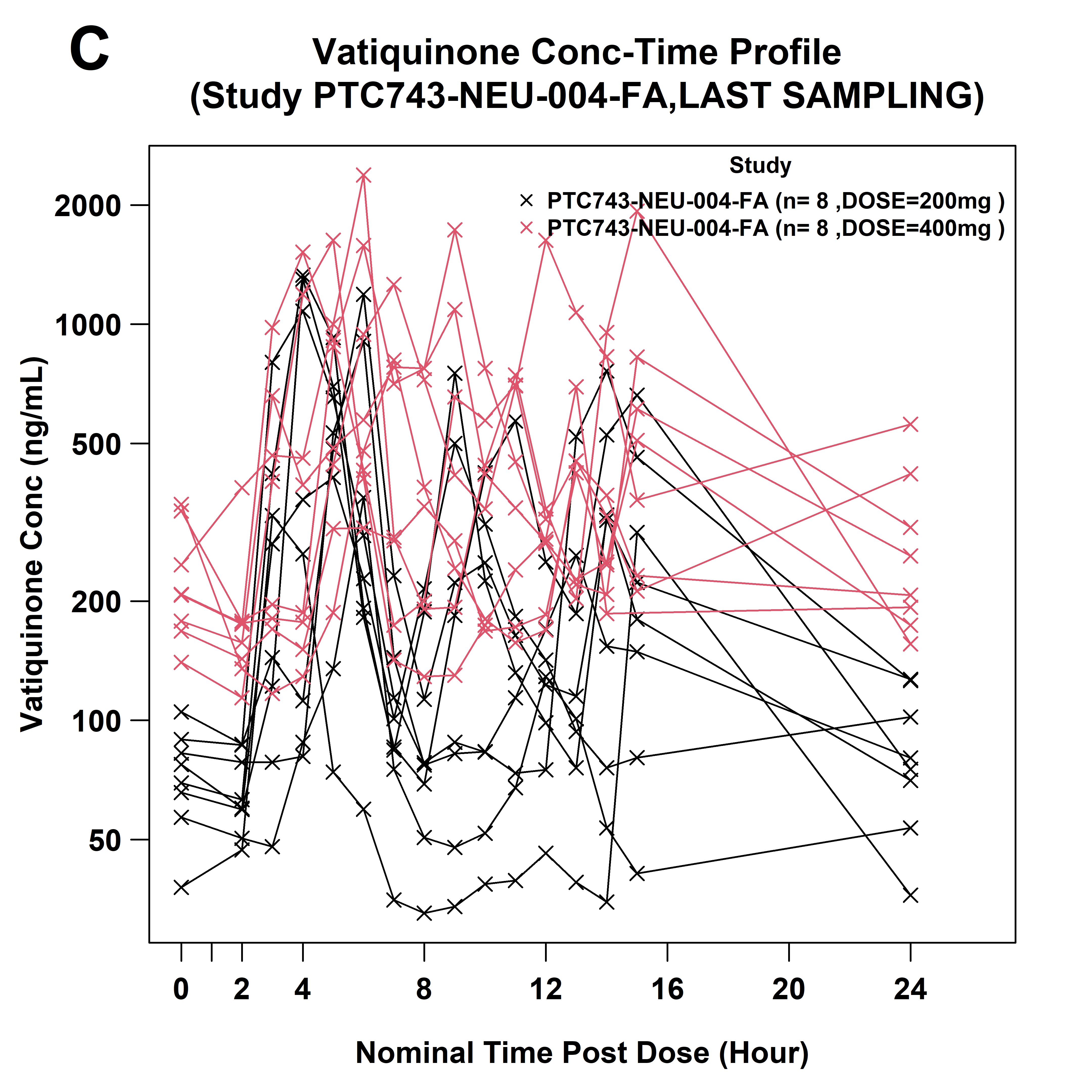

Supplement: Supplementary file 1 [file pharmaceuticals-18-01339-s001.zip › Figure S3C.png]

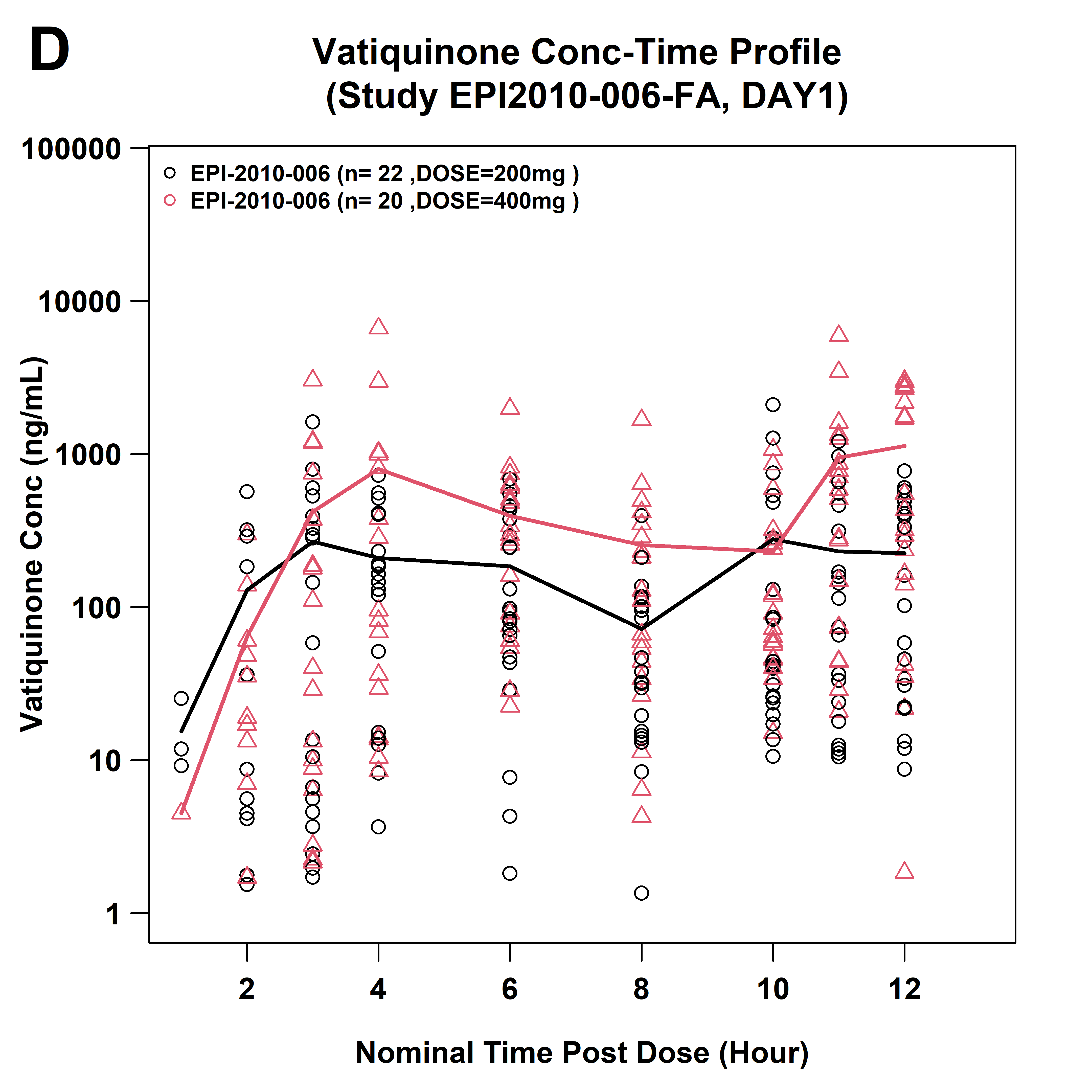

Supplement: Supplementary file 1 [file pharmaceuticals-18-01339-s001.zip › Figure S3D.png]

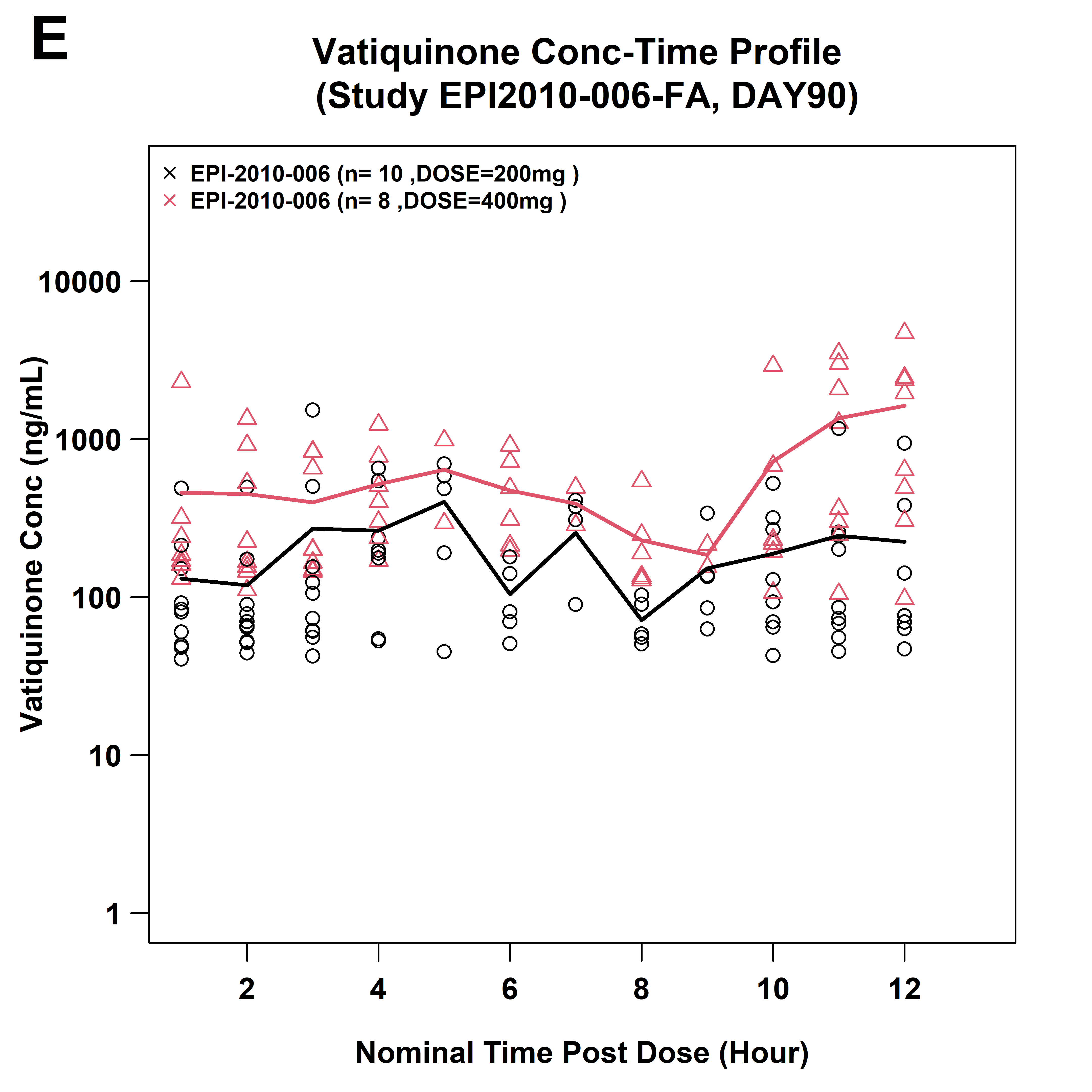

Supplement: Supplementary file 1 [file pharmaceuticals-18-01339-s001.zip › Figure S3E.png]

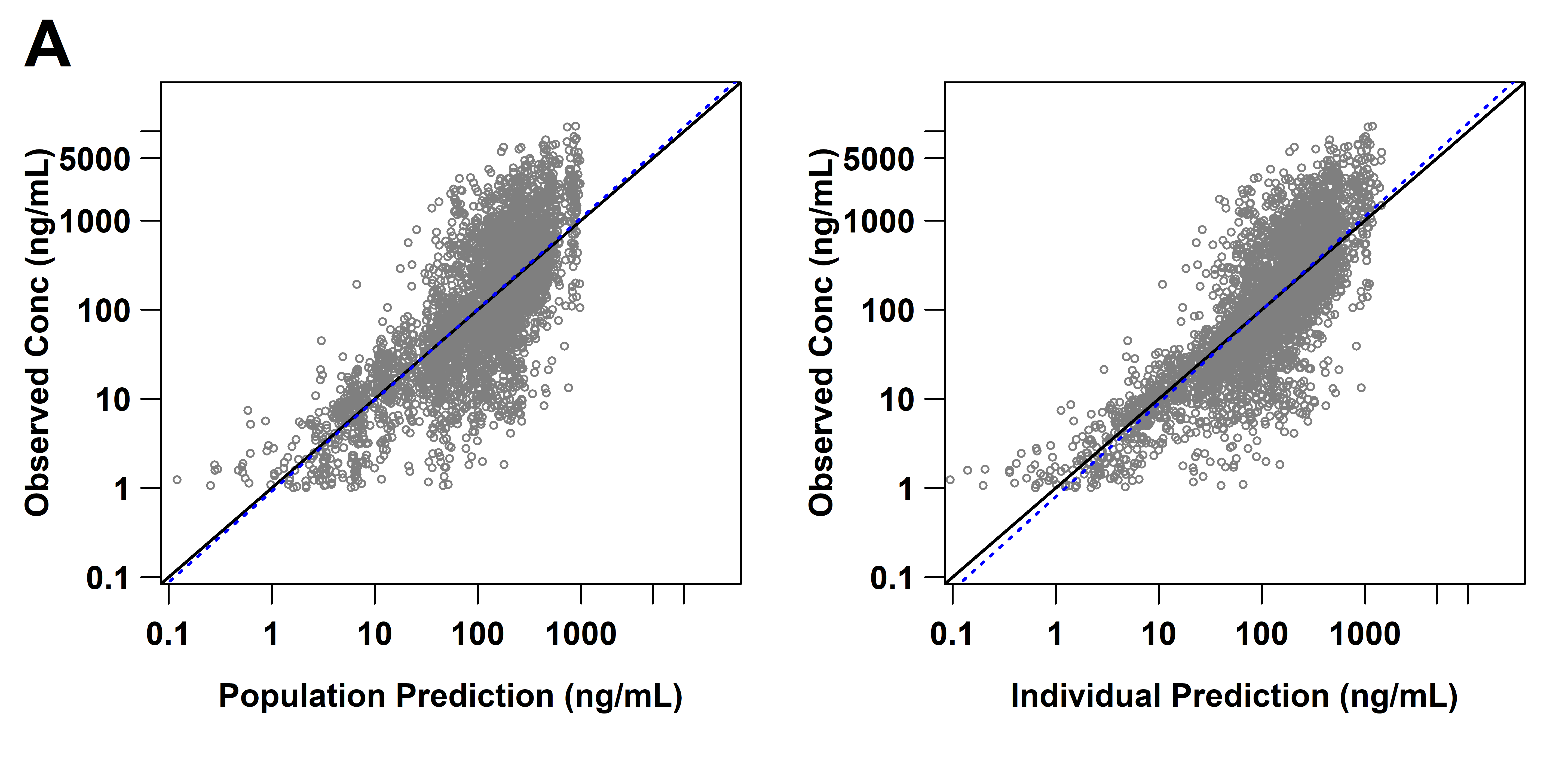

Supplement: Supplementary file 1 [file pharmaceuticals-18-01339-s001.zip › Figure S4A.png]

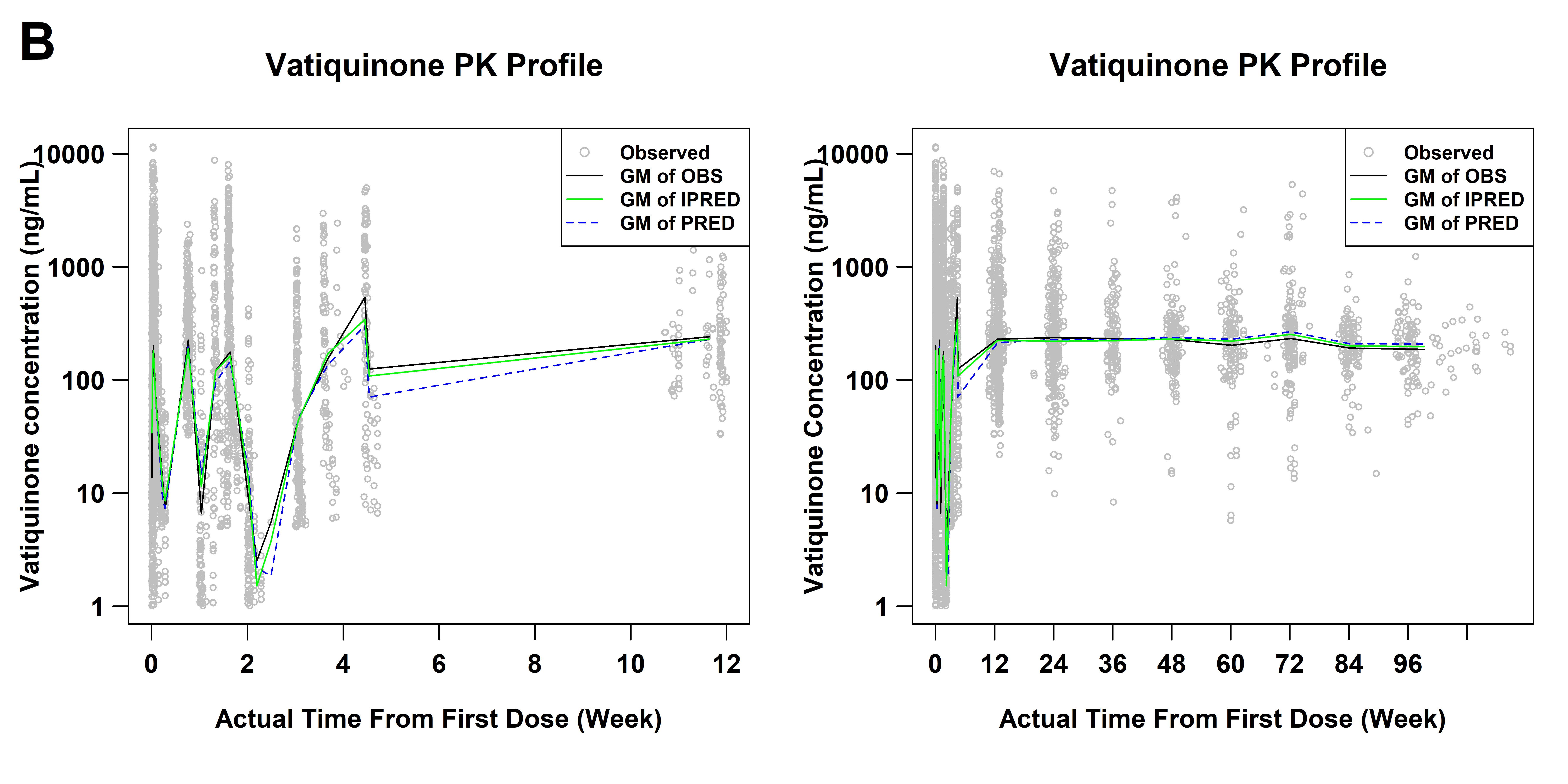

Supplement: Supplementary file 1 [file pharmaceuticals-18-01339-s001.zip › Figure S4B.png]

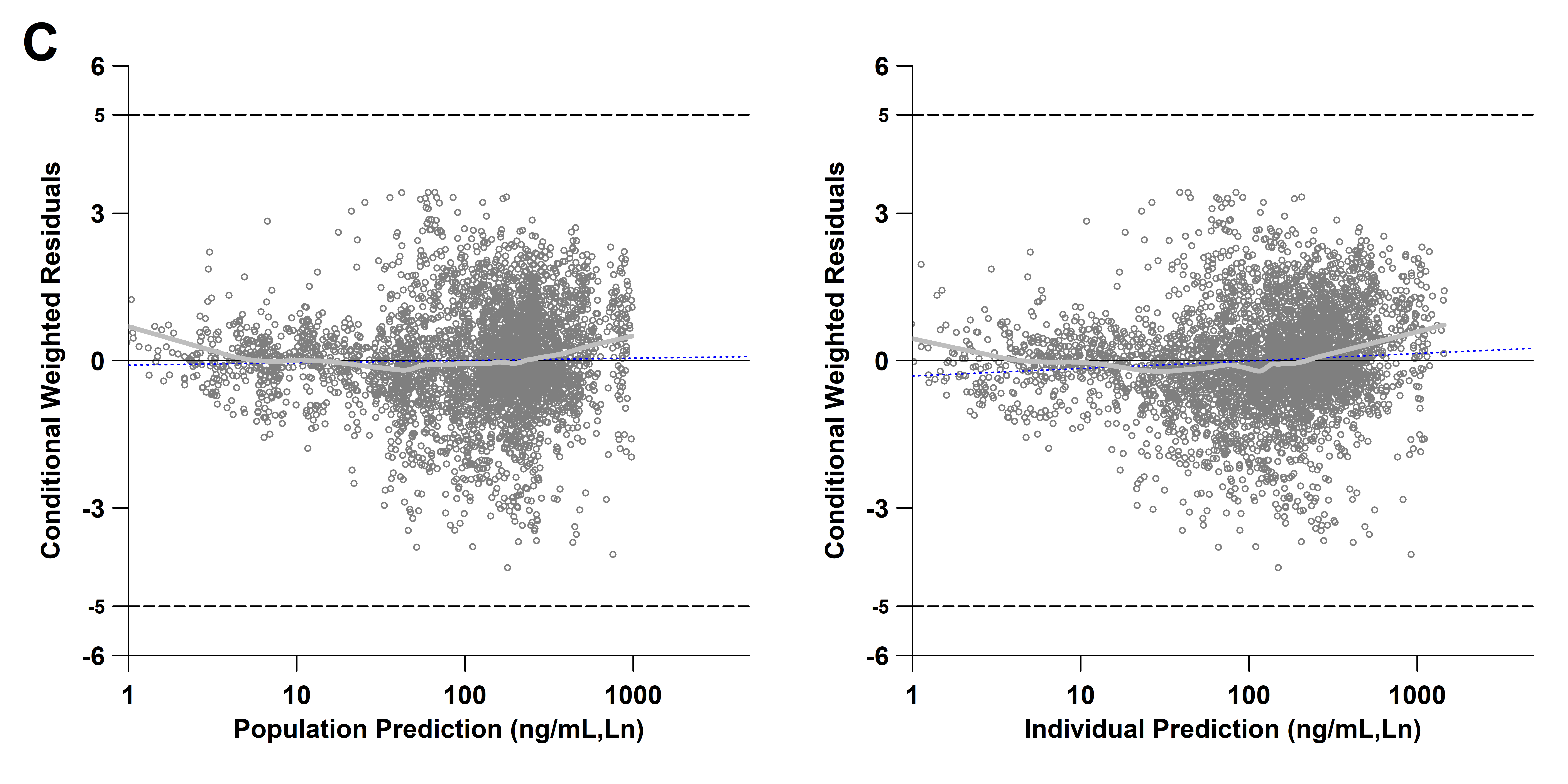

Supplement: Supplementary file 1 [file pharmaceuticals-18-01339-s001.zip › Figure S4C.png]

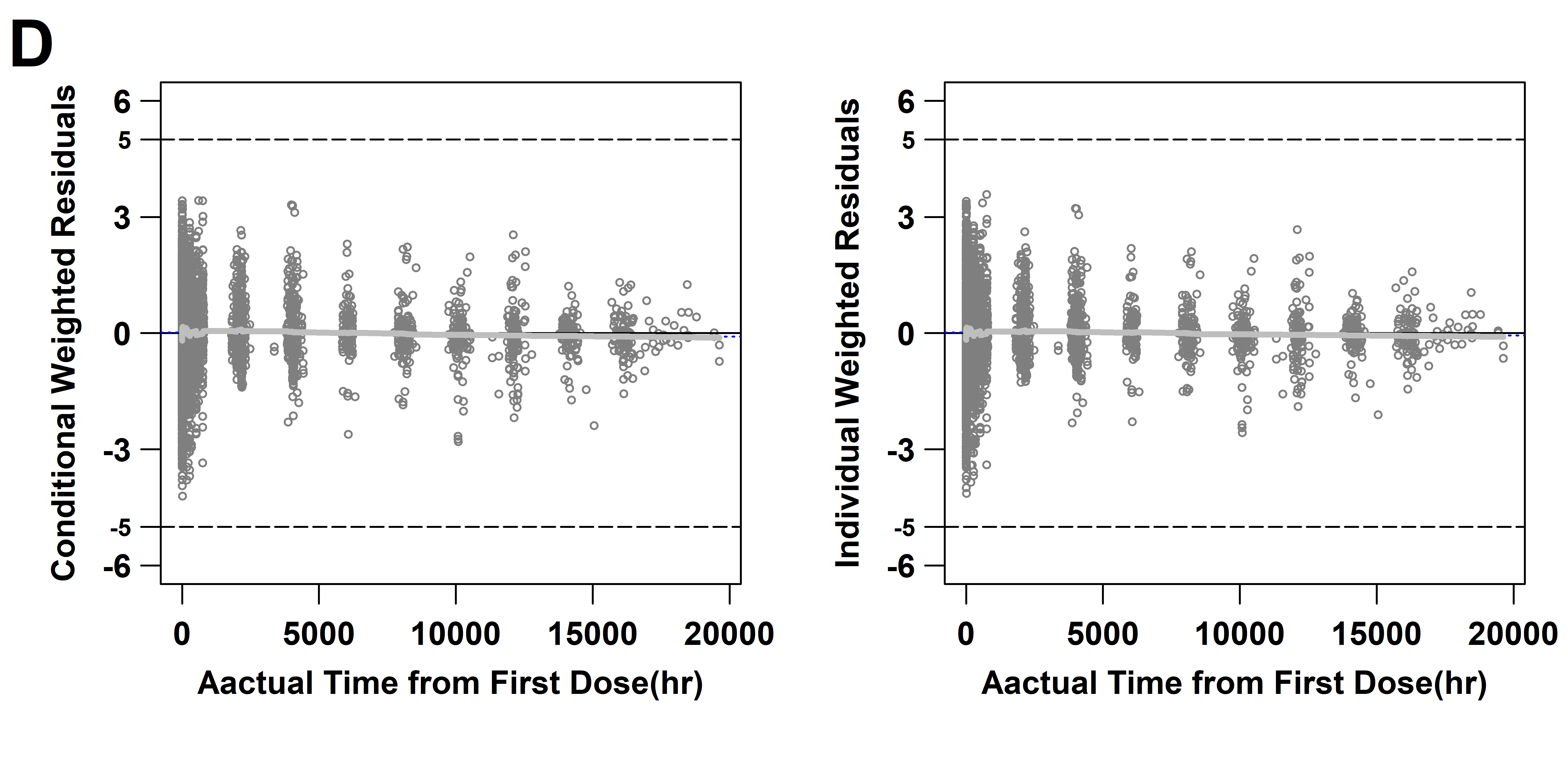

Supplement: Supplementary file 1 [file pharmaceuticals-18-01339-s001.zip › Figure S4D.png]

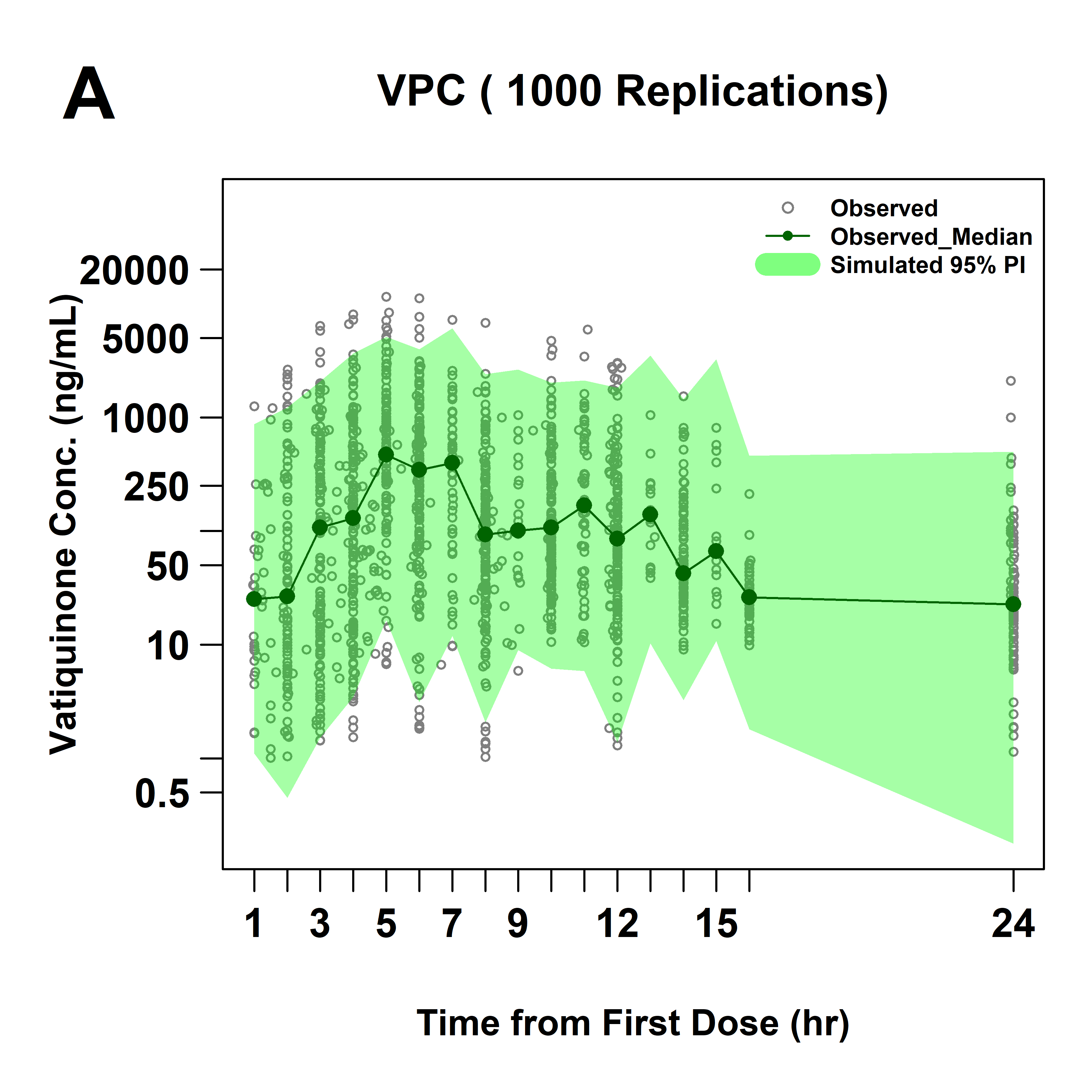

Supplement: Supplementary file 1 [file pharmaceuticals-18-01339-s001.zip › Figure S5A.png]

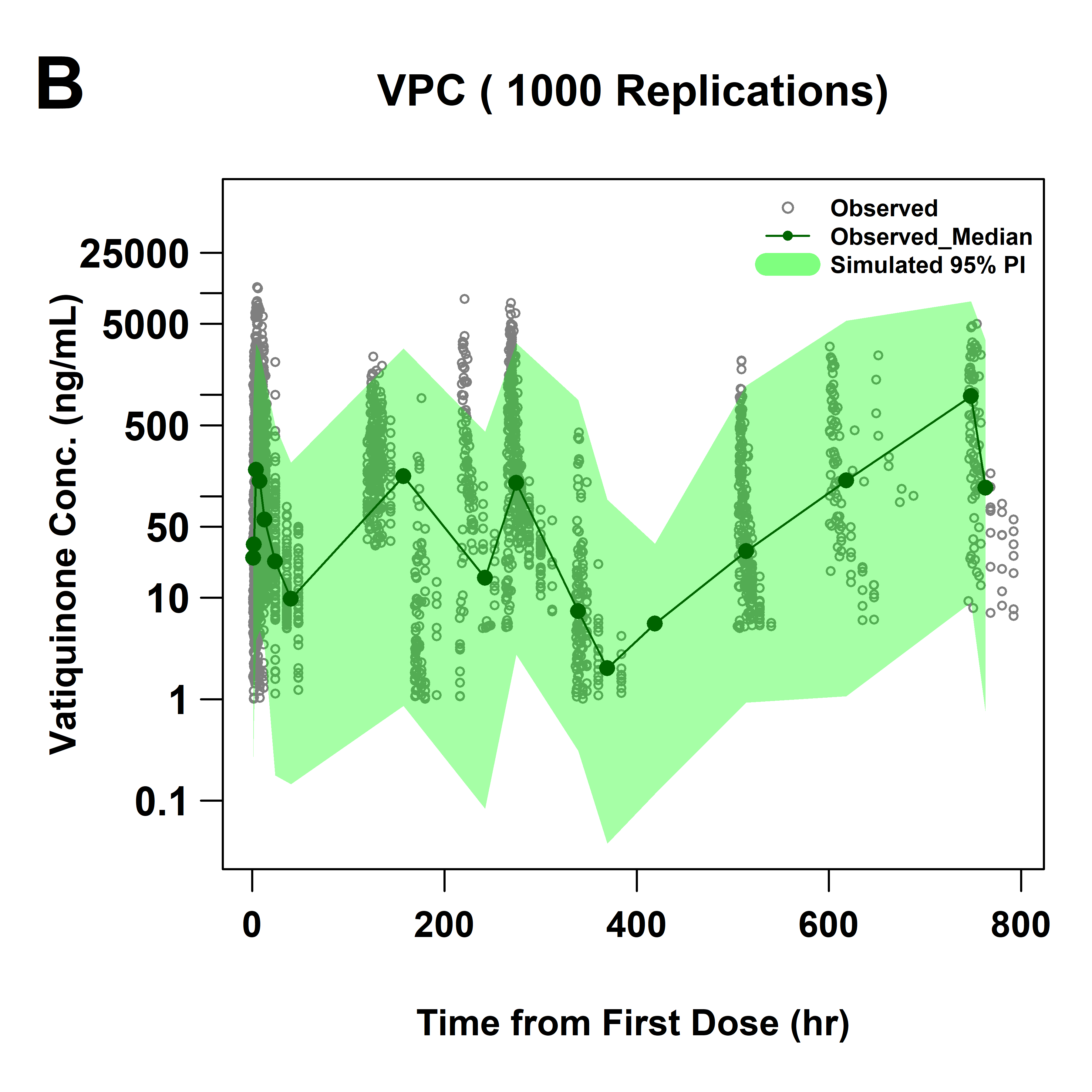

Supplement: Supplementary file 1 [file pharmaceuticals-18-01339-s001.zip › Figure S5B.png]

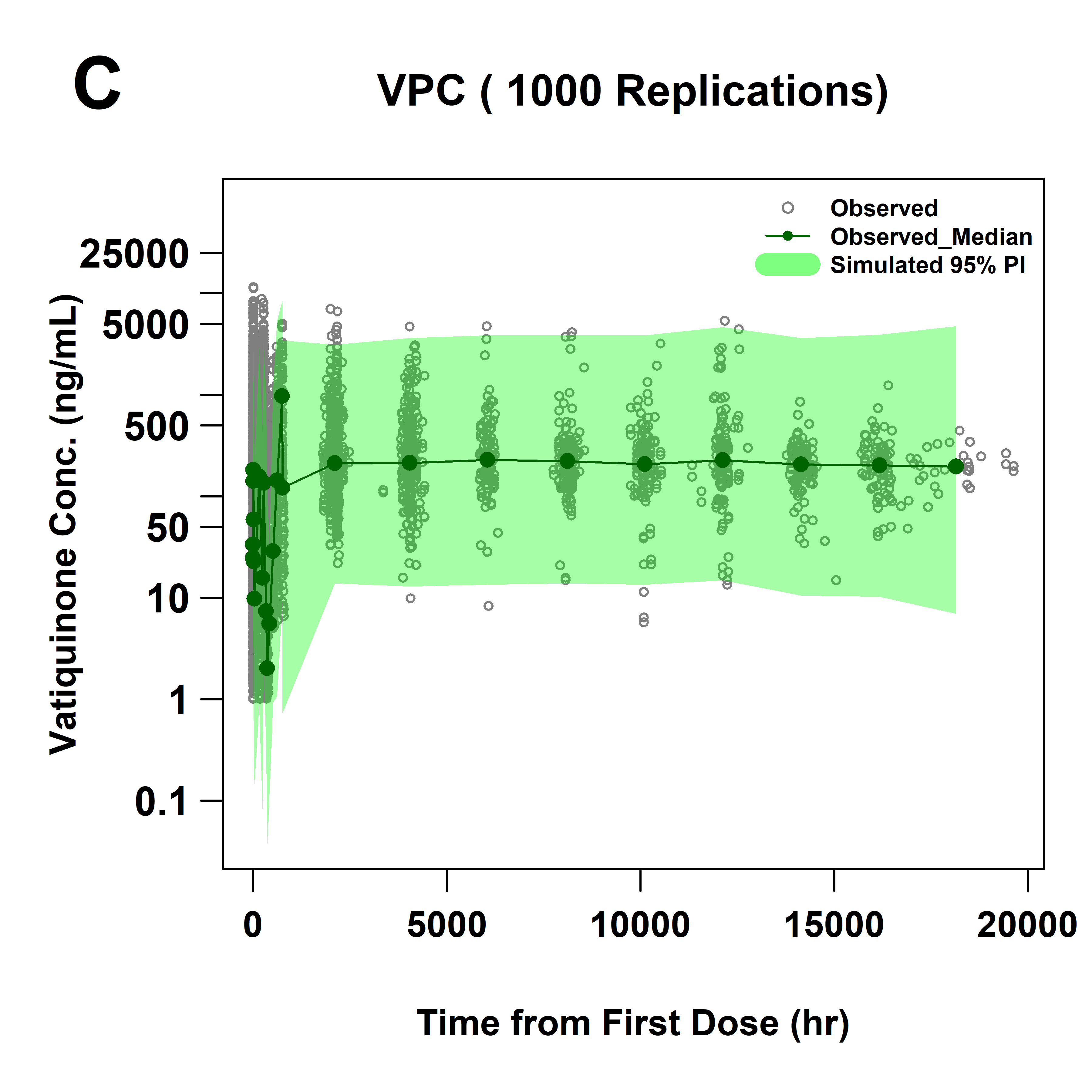

Supplement: Supplementary file 1 [file pharmaceuticals-18-01339-s001.zip › Figure S5C.png]

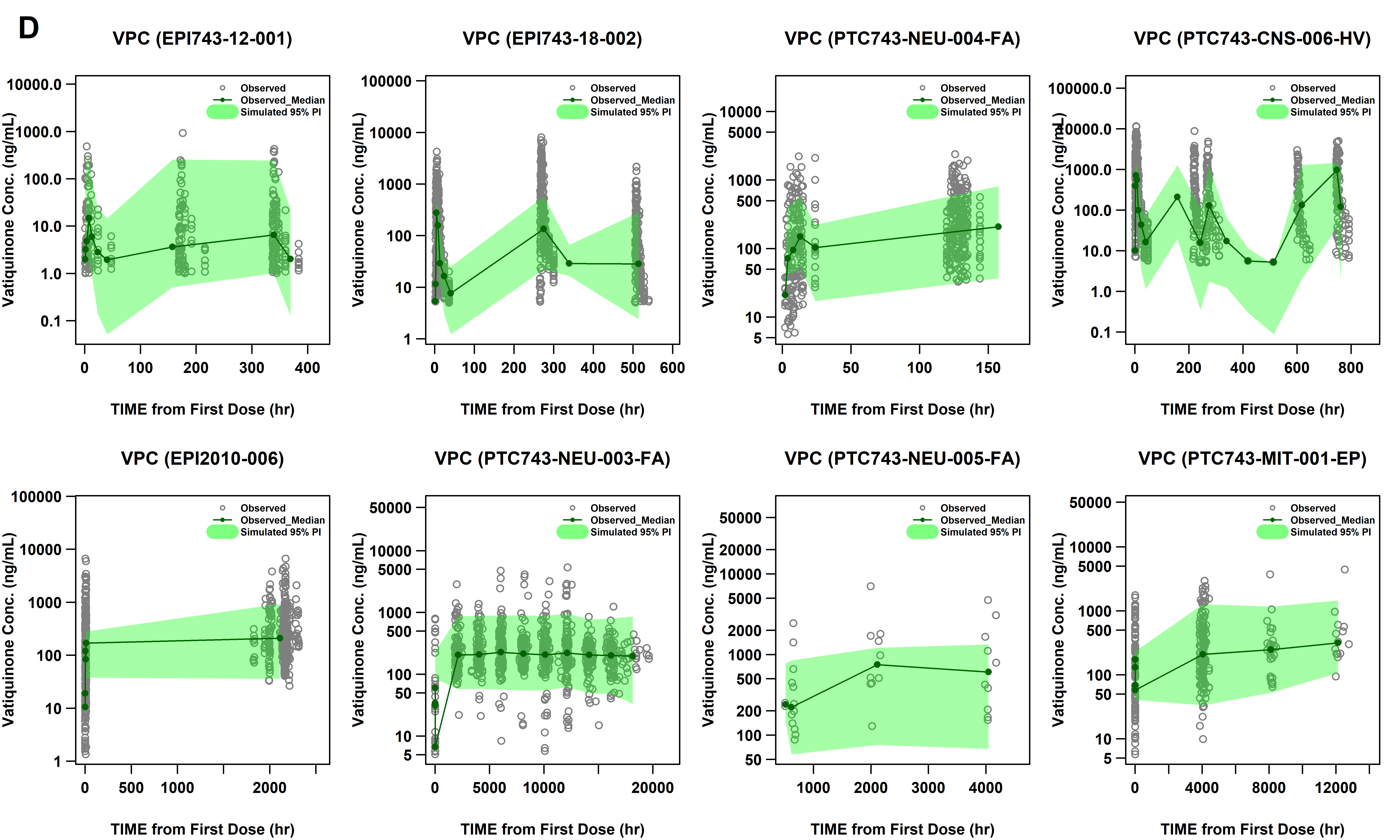

Supplement: Supplementary file 1 [file pharmaceuticals-18-01339-s001.zip › Figure S5D.png]
